# Neurotrophin receptor tyrosine kinases regulated with near-infrared light

**DOI:** 10.1038/s41467-019-08988-3

**Published:** 2019-03-08

**Authors:** Anna V. Leopold, Konstantin G. Chernov, Anton A. Shemetov, Vladislav V. Verkhusha

**Affiliations:** 1 Medicum, Faculty of Medicine, University of Helsinki, Helsinki, 00290 Finland; 2 Department of Anatomy and Structural Biology, and Gruss-Lipper Biophotonics Center, Albert Einstein College of Medicine, Bronx, NY 10461 USA

## Abstract

Optical control over the activity of receptor tyrosine kinases (RTKs) provides an efficient way to reversibly and non-invasively map their functions. We combined catalytic domains of Trk (tropomyosin receptor kinase) family of RTKs, naturally activated by neurotrophins, with photosensory core module of DrBphP bacterial phytochrome to develop opto-kinases, termed Dr-TrkA and Dr-TrkB, reversibly switchable on and off with near-infrared and far-red light. We validated Dr-Trk ability to reversibly light-control several RTK pathways, calcium level, and demonstrated that their activation triggers canonical Trk signaling. Dr-TrkA induced apoptosis in neuroblastoma and glioblastoma, but not in other cell types. Absence of spectral crosstalk between Dr-Trks and blue-light-activatable LOV-domain-based translocation system enabled intracellular targeting of Dr-TrkA independently of its activation, additionally modulating Trk signaling. Dr-Trks have several superior characteristics that make them the opto-kinases of choice for regulation of RTK signaling: high activation range, fast and reversible photoswitching, and multiplexing with visible-light-controllable optogenetic tools.

## Abstract (web summary)

Optical control over receptor tyrosine kinase (RTK) activity can find application in both basic biology and synthetic biology. Here the authors combine the photosensory module of DrBphP bacterial phytochrome with the Trk family of RTKs to obtain Trks that are reversibly switchable with near-infrared and far-red light.

## Introduction

Efficient and selective regulation of receptor tyrosine kinase (RTK) activity is necessary to study a variety of cell signaling pathways in norm and pathology. For quite a while, chemical inhibitors helped to dissect RTK signaling; however, they stalled on the specificity limitation: even most specific of them simultaneously inhibit several RTKs of the same family, making it hard to discern their biological effects. Other chemical approaches, such as bump-and-hole strategy^1^ and chemical dimerizers, played an essential role in RTK studies too, yet have a limited ability to control cell signaling with sufficient spatiotemporal precision. An emerging field of optical regulation of protein kinase activities seeks to address these drawbacks and overcome specificity and spatiotemporal resolution issues at once^2^.

Many of the developed opto-kinases provide possibility for rapid and transient activation of RTK activity, with activation rates higher than that for growth factors regulating kinase activity. The first optically regulated RTKs were developed by Chang et al.^3^ by fusing catalytic kinase domains of tropomyosin receptor kinases (Trks) to the light-responsive photolyase homology region of cryptochrome 2 (CRY2)^3^. Several other opto-kinases based on photosensitive moieties of light-oxygen-voltage-sensing (LOV) domain^4^ and cobalamin-binding domain (CBD)^5^ regulated by blue (LOV) and green (CBD) light were developed too. Upon illumination with light of an appropriate wavelength, the photosensitive domains undergo monomerization–dimerization transitions resulting in reversible activation of opto-kinases. Recently, Zhou et al.^6^ reported opto-kinases with photosensitive moieties of a reversibly switchable fluorescent protein pdDronpa. They are cyan and blue light sensitive, and undergo instant reversible activation/inhibition by steric caging/uncaging of kinase units between two linked pdDronpa proteins. However, all available opto-kinases are regulated with visible light and, therefore, cannot be multiplexed with common fluorescent proteins and biosensors because their fluorescence excitation will simultaneously cause the opto-kinase activation^2^. Engineering of opto-kinases that would enable spectral multiplexing remains a challenge, and photoreceptor domains regulated by far-red (FR) and near-infrared (NIR) light present a promising option to address it^7^.

**Fig. 1:**
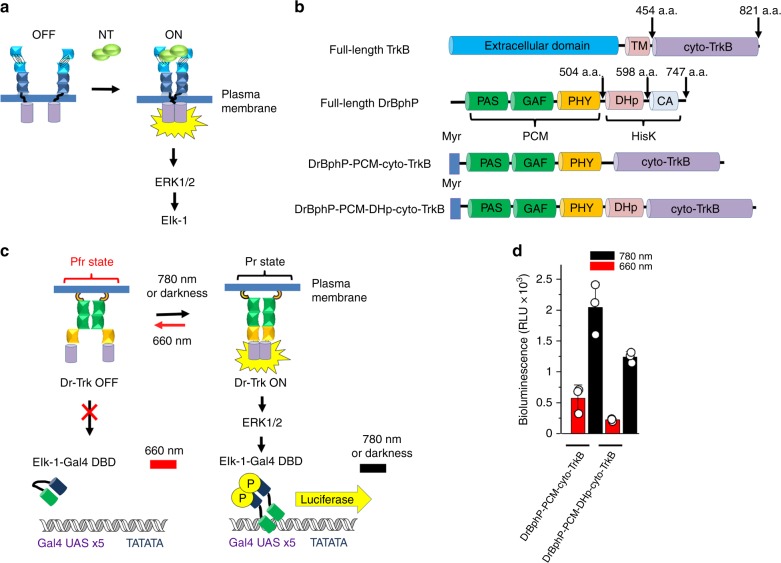
Design and initial screening of DrBphP-PCM kinase fusions. **a** Activation of receptor tyrosine kinases (RTKs) by dimerization upon binding of a growth factor ligand. **b** Schematically depicted structures of the full-length TrkB, DrBphP, and developed for initial screening DrBphP-PCM-cyto-Trk fusion constructs. **c** Scheme of luciferase assay for kinase activity. The system consists of the reporter plasmid, pFR-Luc, where firefly luciferase expression is controlled with the synthetic promoter, containing 5× tandem repeats of the yeast UAS GAL4 binding sites, and the transactivator plasmid pFA-Elk-1. In the transactivator plasmid, the activation domain of the Elk-1 is fused with the yeast GAL4 DNA binding domain (DBD). Under 780 nm light, DrBphP-PCM-cyto-Trk is active, which results in the activation of the MAPK/ERK pathway. The phosphorylated Elk-1-GAL4-DBD fusion dimerizes, binds to 5× UAS, and activates transcription of firefly luciferase. Under 660 nm light, DrBphP-PCM-cyto-Trk is inactive, MAPK/ERK pathway (mitogen-activated protein kinase/extracellular signal-regulated kinase) is inhibited, and luciferase expression is switched OFF. **d** Luciferase assay of initial DrBphP-PCM-cyto-Trk constructs in PC6-3 cells. PC6-3 cells were co-transfected with the pCMVd2-DrBphP-PCM-cyto-Trk, pFR-Luc, and pFA-Elk-1 plasmid mixture (1:100:5), and 6 h after transfection, culture medium was replaced with serum-starving one. Cells were grown for additional 30 h under 780 nm or 660 nm light (both 0.5 mW cm^−2^), lysed, and analyzed for luciferase activity. Error bars represent s.d., *n* = 3 experiments

RTKs are transmembrane receptors comprising a single hydrophobic transmembrane-spanning domain (TM), an extracellular ligand-binding N-terminal region, and a C-terminal cytoplasmic region. The cytoplasmic region, in turn, comprises the juxtamembrane (JM) and catalytic kinase domains. JM domain contains amino acid motifs serving as docking sites for various signaling molecules and plays an essential role in the regulation of RTK activity. In a traditional model of RTK activation, ligand binding induces dimerization of RTK followed by a transphosphorylation of the catalytic kinase domains and RTK activation (Fig. 1a). An increasing number of recent studies demonstrated that RTKs, including TrkA and TrkB, exist as preformed inactive dimers^10^. These findings suggest that RTK activation could be seen as merely a ligand-induced conformational rearrangement of the pre-existing dimers. We hypothesized that the conformational changes accompanying ligand binding could be induced with the help of a light-sensitive dimeric protein fused to the cytoplasmic domains of an RTK, instead of its extracellular domains.

For this, we examined an ability of the *Deinococcus radiodurans* bacterial phytochrome DrBphP^11^ fused to cytoplasmic domains of RTK to modulate kinase activity under illumination with FR (640–680 nm) and NIR (740–780 nm) light. Similar to other canonical bacterial phytochromes^7–10^, DrBphP forms a head-to-head parallel dimer and consists of an N-terminal photosensory core module (PCM) and C-terminal histidine-kinase (HK) domain, connected by a long α-helix (Fig. 1b and Supplementary Fig. 1a). A PCM is the light-sensitive moiety consisting of PAS (Per/Arndt/Sim), GAF (cGMP phosphodiesterase/adenyl cyclase/FhlA), and PHY (phytochrome specific) domains. A HK domain consists of DHp (dimerization histidine phototransfer) and CA (catalytic ATP binding) subdomains (Fig. 1b and Supplementary Fig. 1b). After absorption of light, a biliverdin IXα chromophore (BV) located in the pocket of the GAF domain undergoes *Z-E* transition that induces structural changes propagating from the PCM to the HK domain^8–10^. Under NIR light, DrBphP adopts a Pr state in which the PHY and HK domains of the DrBphP dimer are close to each other. Absorption of FR light converts DrBphP into a Pfr state in which the PHY and HK domains come apart. The Pr-Pfr light-induced transitions are fully reversible (Supplementary Fig. 1a)^11^.

TrkA and TrkB kinases play roles in both norm and pathology^11,12^. The TrkA and TrkB membrane receptors are important for the functioning and survival of neurons and are the most abundant receptors in the mammalian central nervous system. They are activated upon binding of neurotrophic growth factors, termed neurotrophins, such as nerve growth factor (NGF) and brain-derived neurotrophic factor, which trigger the MAPK/ERK (mitogen-activated protein kinase/extracellular signal-regulated kinase), PI3K (phosphoinositide 3-kinase)/Akt, and PLCγ (phosphoinositide phospholipase Cγ) pathways. Also, the cytoplasmic Trk isoforms were identified in several tumor types. They represent oncogenic fusions of the Trks’ catalytic domains with tropomyosin and other proteins. Cytoplasmic isoforms are mainly involved in the MAPK/ERK activation^13^.

Here, we designed and characterized TrkA and TrkB opto-kinases consisting of fusion constructs of the DrBphP’s PCM (DrBphP-PCM) with the TrkA and TrkB kinase domains. The developed FR–NIR light-controllable TrkA and TrkB opto-kinases that were applied to activate Elk-1, CREB, and c-Jun signaling. We then compared ability of FR light and chemical inhibitors to downregulate the activity of the MAPK/ERK pathway. We further showed that the developed opto-kinases reversibly regulate the PI3K pathway, calcium signaling, induce neurite outgrowth in PC6-3 cells, and activate MAPK/ERK signaling in cells implanted in mice. Moreover, we found that the TrkA-based opto-kinase were able to induce apoptosis in neuroblastoma and glioma cells but not in other cell types, including neurons. Lastly, we combined the FR–NIR opto-kinases with the blue-light-activatable LOV2-based protein targeting system, validating spectral multiplexing of two optogenetic tools in a cell.

## Results

### Design and screening of FR–NIR opto-kinase variants

We initially made two constructs by fusing DrBphP-PCM and DrBphP-PCM-DHp truncated variants of DrBphP with the cytoplasmic (JM and catalytic kinase) domains of TrkB (cyto-TrkB) (Fig. 1b and Supplementary Fig. 1). The resulting chimeric proteins were anchored to the plasma membrane via N-terminal myristoylation (myr) signal. We next tested their ability to affect the MAPK/ERK cascade (Fig. 1a) in a light-dependent manner using a PathDetect trans-reporting system (Fig. 1c and Methods). Co-transfection of the DrBphP fusions with transactivator and reporter plasmids into PC6-3 cells enabled light-dependent regulation of the MAPK/ERK pathway. Activation of DrBphP fusions in these cells triggers production of luciferase (Fig. 1c). Indeed, PC6-3 cells grown under 780 nm light demonstrated 4–5-fold higher luciferase expression than the cells grown under 660 nm light (Fig. 1d). Because DrBphP-PCM-cyto-Trk exhibited higher luciferase signal than DrBphP-PCM-DHp-cyto-Trk, we continued working with it.

In the Pfr state induced by FR illumination, the PHY domains of the dimeric DrBphP-PCM are separated by ~35 Å gap, whereas NIR light causes photoconversion from Pfr to Pr state, in which they approach each other (Supplementary Fig. 1a)^14^. Most likely, the similar structural change occurs in DrBphP-PCM-cyto-TrkB chimera upon the Pfr to Pr photoconversion. This leads to the strong spatial separation of the TrkB domains attached to the DrBphP-PCM in the Pfr state and their spatial interaction in the Pr state (Fig. 1c and Supplementary Fig. 1a).

We hypothesized that addition of a rigid linker between the PHY domain and intracellular TrkB domain could increase the distance between the kinase domains in the formed dimer in the Pfr state and, thus, diminish background kinase activity in the Pfr state. To test this hypothesis, we inserted different numbers of A(EAAAK)_n_A α-helical repeats^12^ between DrBphP-PCM and cyto-TrkB (Supplementary Fig. 2) and tested the ability of the resulting constructs to regulate MAPK/ERK pathway in a light-dependent manner (Fig. 1c). Addition of one, two, or three α-helical repeats slightly increased the luciferase reporter signal in Pr state without affecting background level in Pfr state, whereas a linker with four repeats decreased background kinase activity by ~3-fold while leaving the Pr state-specific activity unchanged (Supplementary Fig. 2c). Further increase of the linker length proved to be suboptimal: the linker with five α-helical repeats reduced the activity of DrBphP-PCM-cyto-TrkB fusion in both Pr and Pfr states (Supplementary Fig. 2c). The same approach applied to DrBphP-PCM-cyto-TrkA chimera again demonstrated that the linker with four helical repeats had an optimal balance between background and specific kinase activities (Supplementary Fig. 2b, d). These optimal fusion constructs with the rigid linker containing four α-helical repeats were termed Dr-TrkA and Dr-TrkB and used in further experiments.

### Initial characterization of Dr-Trk opto-kinases

It has been previously shown that activation of CRY- and LOV-based opto-kinases occurs quite fast^3,13^. To confirm the rapid autophosphorylation of Dr-Trks upon light activation, we stimulated cells with NIR light and analyzed their lysates with anti-phospho-Trk and anti-phospho-ERK antibodies. We found that an increase in the level of phosphorylated Dr-TrkA (Supplementary Fig. 3) and phosphorylated ERK (Supplementary Fig. 4) occurs already within 5 min of NIR illumination. Similar fast activation was observed for stimulation of neuroblastoma cells with neurotrophins^3^ that bind and activate endogenous Trk receptors.

To rule out possible variation of Dr-Trk protein stability under different light, we quantified its expression after prolonged illumination and found that neither FR nor NIR light affects the Dr-Trk protein levels (Supplementary Fig. 5).

Moreover, we found that common white light of luminescent lamps did not activate Dr-Trks in PC6-3 and HeLa cells. In contrast, darkness caused Dr-Trk activation, but 20–40% lower than NIR light (Supplementary Fig. 6). These results suggest that Dr-Trks resemble the photophysical properties of parental DrBphP, which has the substantially higher Pr-to-Pfr photoconversion efficiency under FR light than that from the Pfr state to the Pr state under NIR light, and exhibits considerably slower conversion from the Pfr state to the Pr ground state in darkness (thermal relaxation) than under NIR light^11^.

We then verified that neither FR nor NIR light affected signaling of endogenous TrkA and exogenous membrane-anchored myr-GST-cyto-TrkB fusion dimerized via a glutathione *S*-transferase (GST) protein^15^ (Supplementary Fig. 7). We also confirmed the plasma membrane localization of Dr-Trks in PC6-3 and HeLa cells (Supplementary Fig. 8).

### Activation of Elk-1, CREB, and c-Jun signaling with Dr-Trks

**Fig. 2:**
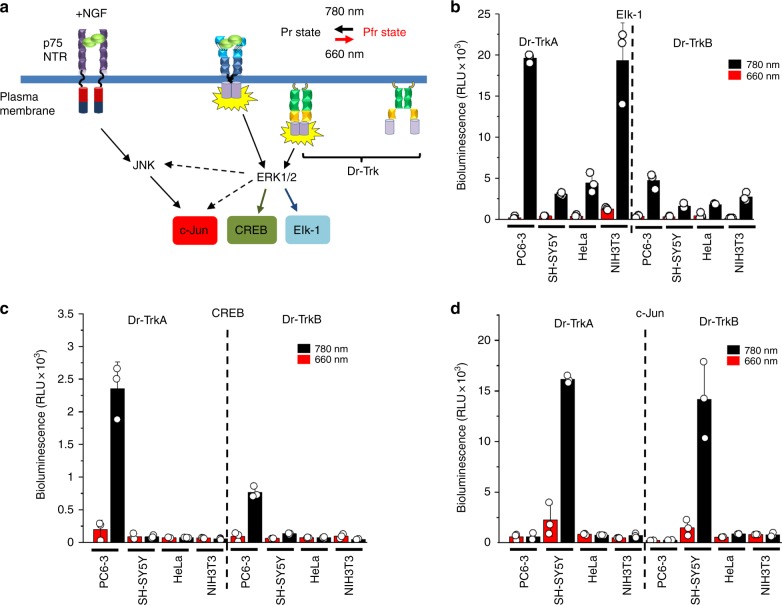
Light-dependent activation of Elk-1, CREB, and c-Jun in various cell lines. **a** The scheme of the activation of Elk-1, CREB, and c-Jun transcription factors upon activation of the receptor tyrosine kinases (RTKs) and Dr-Trks. **b** Luciferase assay for Elk-1-dependent transcription in various cell lines. PC6-3, HeLa, SH-SY5Y, and NIH3T3 cells were plated in 24-well plate and incubated with the pCMVd2-Dr-Trk, pFR-Luc, and pFA-Elk-1 plasmid mixture (mass ratio 1:100:5 for PC6-3 or 1:100:1 for other cell lines) for 6 h. Medium then was replaced with serum-starving one, cells were grown for additional 30 h under 780 nm or 660 nm light (both 0.5 mW cm^−2^), lysed, and analyzed for luciferase activity. **c** Luciferase assay for CREB-dependent transcription. The indicated cell lines were co-transfected with the pCMVd2-Dr-Trk, pFR-Luc, and pFA-CREB plasmid mixture (mass ratio 1:100:5 for PC6-3 or 1:100:1 for other cell lines), grown for 30 h under 780 nm or 660 nm light (both 0.5 mW cm^−2^), lysed, and analyzed for luciferase activity. **d** Luciferase assay for c-Jun-dependent transcription. Cells were co-transfected with the pCMVd2-Dr-Trk, pFR-Luc, and pFA-c-Jun plasmid mixture (mass ratio 1:100:5 for PC6-3 cells or 1:100:1 for other cell lines), grown as above, and analyzed for luciferase activity. Error bars represent s.d., *n* = 3 experiments

A prosurvival MAPK/ERK pathway is a major downstream signaling pathway activated by Trks^16^. In different cell types, its activation outcome ranges from proliferation to differentiation. The main downstream targets of Trks are the transcription activator Elk-1 and, in some cells, the transcription activator CREB (cAMP response element-binding protein)^17^. In certain cases, prolonged activation of the MAPK/ERK also causes upregulation of the stress-related transcription factor c-Jun, which is a downstream target of the JNK/SAPK pathway (Fig. 2a)^18^.

Activation of Elk-1 by Dr-Trks was tested in several different cell types: PC6-3 pheochromocytoma (derived from a peripheral nervous system tumor), epithelial HeLa cells, SH-SY5Y neuroblastoma, and NIH3T3 fibroblasts. Elk-1 activity was assessed by co-transfection of Dr-Trks with the pFR-Luc reporter and pFA-Elk-1 transactivator plasmids. In all cell types, Elk-1 activity was upregulated by 780 nm NIR and inhibited by 660 nm FR light (Fig. 2b). The highest level of Elk-1 activation was achieved in the PC6-3 cells, where activation by Dr-TrkA and Dr-TrkB by 780 nm light was 33- and 27-fold more efficient as compared to 660 nm light. In SH-SY5Y cells, activation of Elk-1 by Dr-TrkA and Dr-TrkB under 780 nm light was 15- and 6-fold higher than with 660 nm light; in HeLa cells, 21- and 10-fold, and in NIH3T3, both Dr-Trks showed up to 10-fold Elk-1 upregulation. In all cell types, Dr-TrkB activated Elk-1 to a lesser extent than Dr-TrkA (Fig. 2b), presumably due to the earlier reported structural variations, resulting in the different kinetics of their activation and downstream signaling^19^. The similar difference in Elk-1 activation was observed for cytoplasmic cyto-Dr-TrkA and cyto-Dr-TrkB constructs without myristoylation signals (Supplementary Fig. 9).

Dr-Trk activation of two other downstream targets, CREB and c-Jun transcription factors, strongly depended on the cell type. Upregulation of CREB activity by 780 nm light was observed only in PC6-3 cells and was 12- and 6-fold higher than under 660 nm light for Dr-TrkA and Dr-TrkB, respectively (Fig. 2c). Similarly, no activation of c-Jun-dependent transcription was detected in PC6-3, HeLa, and NIH3T3 cells (Fig. 2d), whereas in SH-SY5Y neuroblastoma activation of c-Jun was observed for both Dr-TrkA (6-fold) and Dr-TrkB (10-fold). To check whether Dr-Trk activation causes activation of downstream ERK signaling, we treated SH-SY5Y cells with ERK-specific inhibitor SCH772984. SCH772984 equally inhibited both c-Jun and Elk-1 expression (Supplementary Fig. 10). Likely, the c-Jun upregulation in SH-SY5Y cells was caused by a sustained ERK activation, which is in agreement with the reported connection between the sustained ERK signaling and JNK pathway in neuroblastomas but not in the cells of other origins^18^.

From these experiments, we concluded that the Dr-Trk could efficiently control the Elk-1, CREB, and c-Jun signaling with FR-NIR light in the specific cell types.

### Action of tyrosine kinase inhibitors and FR light on Dr-Trks

**Fig. 3:**
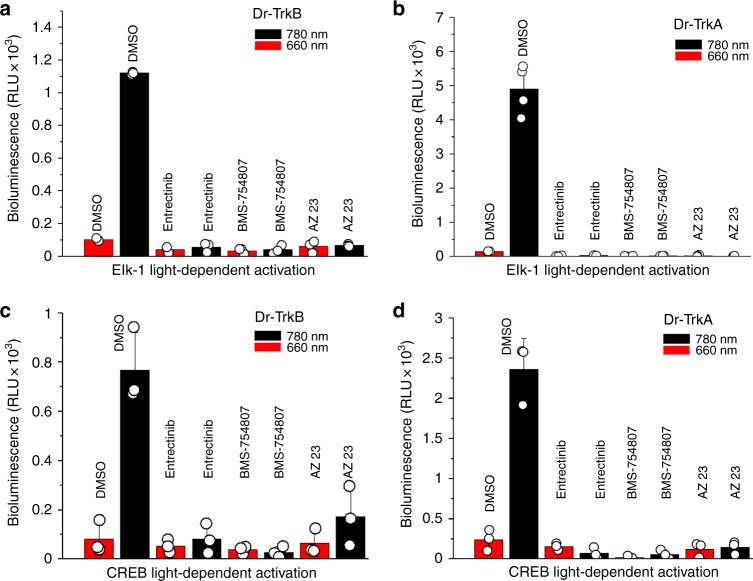
Downregulation of Dr-Trk activity with FR light and kinase inhibitors. **a** Luciferase assay for Elk-1-dependent transcription in PC6-3 cells co-transfected with pCMVd2-Dr-TrkB, pFR-Luc, and pFA-Elk-1 plasmid mixture (1:100:5) and grown for 30 h under 660 nm or 780 nm light (both 0.5 mW cm^−2^). The indicated kinase inhibitors (100 nM) or dimethyl sulfoxide (DMSO) solvent (0.1%) were added to the culture medium. **b** Luciferase assay for Elk-1-dependent transcription in PC6-3 cells co-transfected with pCMVd2-Dr-TrkA, pFR-Luc, and pFA-Elk-1 plasmid mixture and grown under 660 nm or 780 nm light. **c** Luciferase assay for CREB-dependent transcription in PC6-3 cells co-transfected with pCMVd2-Dr-TrkB, pFR-Luc, and pFA-CREB plasmid and grown under 660 nm or 780 nm light in the presence of kinase inhibitors. **d** Luciferase assay for CREB-dependent transcription in PC6-3 cells co-transfected with pCMVd2-Dr-TrkA, pFR-Luc, and pFA-CREB plasmid mixture and grown under 660 nm or 780 nm light in the presence of kinase inhibitors. Error bars represent s.d., *n* = 3 experiments

Chemical regulators of RTK activity are common tools for studies of signaling pathways. To further characterize the Dr-Trk opto-kinases we compared efficiency of FR light and tyrosine kinase inhibitors to suppress the Elk-1 and CREB activities. For this, we have chosen entrectinib and BMS-754807, which inhibit a broad spectrum of tyrosine kinases, and AZ23, which is a specific inhibitor of the Trk family^20^. In PC6-3 cells expressing Dr-TrkA or Dr-TrkB, the 100 nM of entrectinib, BMS-754807, and AZ23 inhibitors decreased the Elk-1-dependent transcription slightly more than FR light for both Dr-Trk constructs (Fig. 3a, b). The larger downregulation of the Elk-1 activity by inhibitors likely resulted from the additional suppression of endogenous RTKs, whereas the FR light inhibited the Dr-Trks only. In the case of CREB transcription factor, all tested inhibitors including the Trk-specific AZ23 and FR light suppressed the CREB-dependent luciferase expression with similar efficiency (Fig. 3c, d). In control experiments we confirmed that the inhibitors did not affect cell viability (Supplementary Fig. 11). We concluded that both Dr-Trk opto-kinases are efficiently inhibited by common tyrosine kinase inhibitors and that the efficiency of their inhibition by FR light is comparable to that by the chemical inhibitors.

### Reversible light-control of PI3K/Akt pathway

**Fig. 4:**
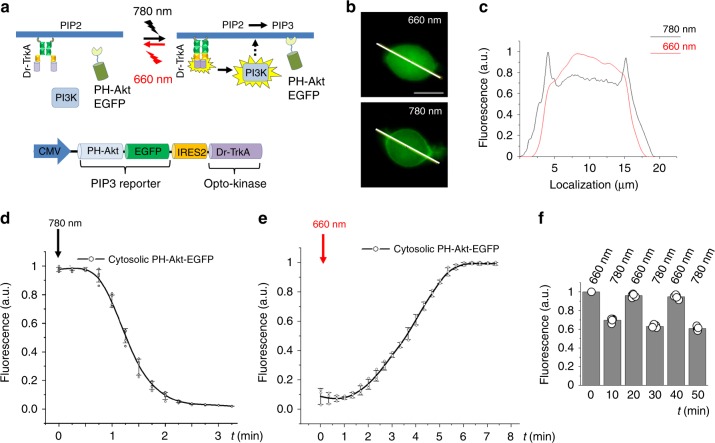
Light-dependent translocation of PH-Akt-EGFP reporter in PC6-3 cells. **a** Top: Dr-TrkA is inactive in cells kept under 660 nm light. The 780 nm light causes activation of the Dr-TrkA and downstream phosphatidylinositol triphosphate (PIP3) kinase. This results in accumulation of PIP3 in the plasma membrane and recruiting of PH-Akt-EGFP reporter from the cytoplasm. Bottom: Schematic drawing of the bicistronic pPH-Akt-EGFP-IRES2-Dr-TrkA plasmid co-expressing PH-Akt-EGFP and Dr-TrkA. **b** Top: Epifluorescence image of the PC6-3 cell transfected with the bicistronic plasmid and kept under 660 nm light. Bottom: Epifluorescence image of the PC6-3 cell kept under 780 nm light for 10 min to reach steady-state Akt transition to the plasma membrane. Scale bar, 10 μm. **c** Steady-state intensity profile of PH-EGFP-Akt fluorescence of the PC6-3 cell kept under 660 nm light (red line) and illuminated by 780 nm light for 10 min (black line). **d** Relative decrease of cytoplasmic PH-Akt-EGFP fluorescence induced by 780 nm light (0.5 mW cm^−2^). **e** Relative increase of cytoplasmic PH-Akt-EGFP fluorescence induced by 660 nm light (0.5 mW cm^−2^). **f** Reversible translocation of the PH-Akt-EGFP reporter between the plasma membrane and cytoplasm in response to 780 nm and 660 nm illumination. Error bars represent s.d., *n* = 3 experiments

RTK activation results in the upregulation of PI3K/Akt signaling and accumulation of phosphatidylinositol triphosphate (PIP3) at the plasma membrane, which could be traced by translocation of a PH-Akt-EGFP sensor from the cytoplasm to the membrane^21^. To determine whether Dr-Trks could optically control the PI3K/Akt pathway, we co-expressed the PH-Akt-EGFP sensor and Dr-TrkA from bicistronic plasmid PH-Akt-EGFP-IRES2-Dr-TrkA in PC6-3 cells (Fig. 4a). The cells kept under FR light had an even cytoplasmic distribution of PH-Akt-EGFP. Illumination with NIR light resulted in the activation of Dr-TrkA and translocation of the PH-Akt-EGFP to the plasma membrane (Fig. 4b, c). A light-induced translocation half-time was 1.25 min (Fig. 4d), which was comparable to a translocation half-time of 2 min for the control PC6-3 cells stimulated with NGF (Supplementary Fig. 12a, b, c). The light activation of the downstream PI3K signaling was reversible with a half-time of PH-Akt-EGFP dissociation from the plasma membrane being 3.5 min (Fig. 4e). The latter kinetics was comparable to that observed for AZ23 inhibitor (3 min) but 4-fold faster than that after the NGF removal in the control cells (Supplementary Fig. 12d, e). While NGF acted on the endogenous TrkA and p75NTR, NIR and FR light affected only Dr-TrkA. The intensity profile of PH-Akt-EGFP sensor, measured by epifluorescence microscopy at the steady-state condition, showed 30–40% change in cytoplasmic fluorescence intensity upon alternating 10 min illumination periods of FR and NIR light (Fig. 4c, f). This indicated that Dr-Trk opto-kinase activity could repeatedly be turned ON and OFF, providing the efficient and reversible activation–inhibition of PI3K/Akt signaling.

### Reversible light-control of calcium signaling

**Fig. 5:**
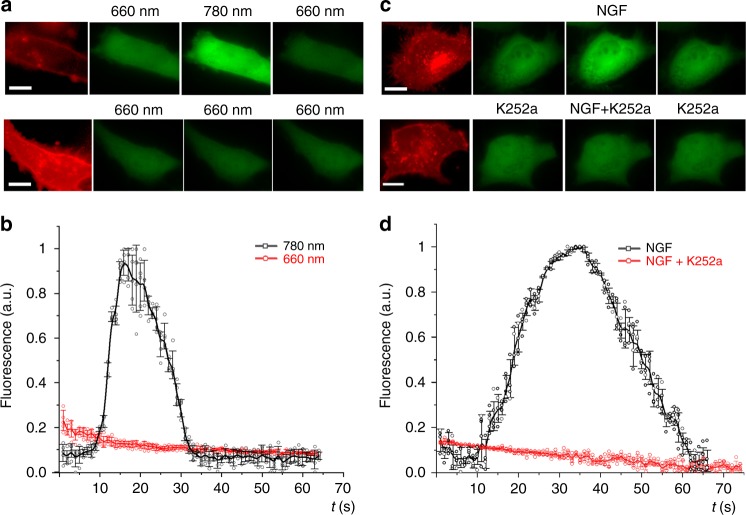
Activation of calcium signaling by Dr-TrkA. **a** Images of HeLa cells co-expressing mCherry-Dr-TrkA and GCaMP6m. Upper row: cells under constant 660 nm light (0.5 mW cm^−2^) were stimulated with 20 s pulse of 780 nm light (0.5 mW cm^−2^). Bottom row: the same cells under constant 660 nm light (0.5 mW cm^−2^). Note the decrease of cellular calcium in the last images. Scale bar, 10 μm. **b** Changes of GCaMP6m fluorescence for Dr-TrkA-expressing cells either stimulated by 780 nm light for 20 s (black line) or kept under 660 nm light (red line). **c** Images of HeLa cells co-expressing TrkA-DsRed2 and GCaMP6m. Upper row: cells before and after addition of 50 ng ml^−1^ nerve growth factor (NGF). Bottom row: cells stimulated with NGF in the presence of 100 nM of K252a. Note the decrease of cellular calcium in the last images. Scale bar, 10 μm. **d** Changes of GCaMP6m fluorescence upon stimulation of the cells with NGF in the absence (black line) or presence of K252a (red line). Error bars represent s.d., *n* = 3 experiments

To characterize activation of calcium-dependent signaling, we co-transfected HeLa cells with plasmids encoding calcium biosensor GCaMP6m and Dr-TrkA and monitored their response to light. Stimulation of the cells with 20 s pulse of NIR light caused an increase of GCaMP6m fluorescence, which then decreased after the light was changed to FR (Fig. 5a, b). In contrast, in the cells illuminated all time with FR light, calcium changes were absent. A calcium spike detected by GCaMP6m fluorescence was also observed in the cells treated with NGF (Fig. 5c, d). The observed calcium spikes were completely inhibited by Trk-specific inhibitor K252a, confirming the direct activation of calcium influxes by Dr-TrkA and endogenous TrkA. The light-induced increase of calcium was very fast, with a half-time of ~7 s, which was 3-fold faster than that for the NGF-stimulated cells (Fig. 5b, d). Similarly, the calcium decrease in the Dr-TrkA cells was more rapid, with a half-time of ~10 s, than in the cells with endogenous TrkA only. We concluded that Dr-TrkA is capable of providing more precise temporal control over calcium-dependent signaling than NGF-activated TrkA receptors.

### Activation of Dr-Trks causes neurite outgrowth in PC6-3 cells

**Fig. 6:**
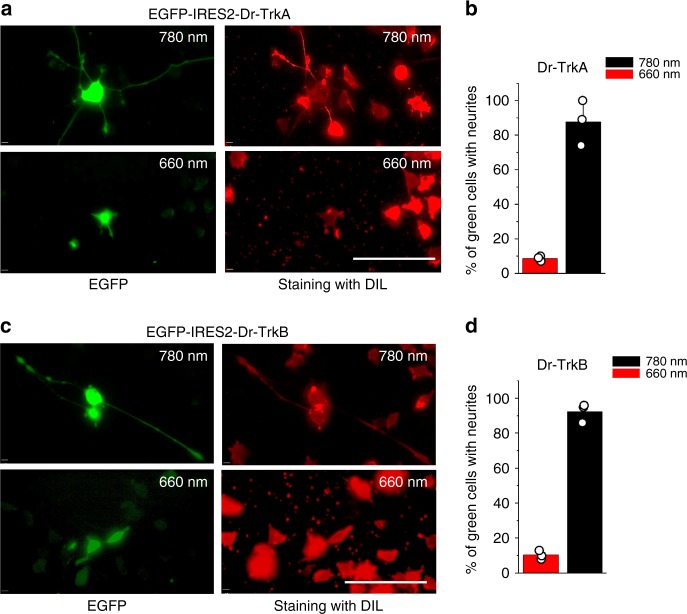
Light-dependent neurite outgrowth in PC6-3 cells. **a** Epifluorescence images of PC6-3 cells transfected with the pEGFP-IRES2-Dr-TrkA plasmid kept for 72 h under either 780 nm (upper panels) or 660 nm (lower panels) light. Before imaging, the cells were fixed and stained with a Dil reagent. Fluorescence images were taken in EGFP (left) and Dil (right) channels. **b** Quantification of cells bearing neurites from the total number of 50 cells transfected with the pEGFP-IRES2-Dr-TrkA plasmid. **c** Epifluorescence images of PC6-3 cells transfected with pEGFP-IRES2-Dr-TrkB plasmid kept for 72 h under either 780 nm (upper panels) or 660 nm (lower panels) light. Before imaging, cells were fixed and stained with a Dil reagent. Fluorescence images were taken in EGFP (left) and Dil (right) channels. **d** Quantification of the cells bearing neurites from the total number of 50 cells transfected with the pEGFP-IRES2-Dr-TrkB plasmid. Error bars represent s.d., *n* = 3 experiments. Scale bar, 100 µm

Treatment of PC6-3 cells with NGF and other neurotrophins results in neurite outgrowth and cell differentiation, whereas incubation with EGF leads to cell proliferation. These opposite effects are caused by sustained and transient activation of MAPK/ERK pathway, respectively^22^. To test an ability of Dr-Trks to simulate the effect of neurotrophins during the long periods of NIR illumination, we constructed the EGFP-IRES2-Dr-Trk bicistronic vectors to allow visualization of cells expressing Dr-Trk by enhanced green fluorescent protein (EGFP) fluorescence. We found that after 72 h of NIR illumination, more than 85% of PC6-3 cells expressing Dr-TrkA or Dr-TrkB developed neurites (Fig. 6), whereas less than 15% of PC6-3 cells developed neurites upon FR illumination. The neurite outgrowth induced by NGF in control PC6-3 cells was apparent already after 24 h of treatment when the Dr-Trk-induced neurites were still undetectable (Supplementary Fig. 13). The Dr-Trk constructs produced longer but less abundant neurites than NGF, which in addition to endogenous TrkA activated the endogenous p75NTR receptor, likely resulting in shorter and more numerous neurites. We concluded that the prolonged periods of the ON state of Dr-Trk opto-kinases can cause the neurite outgrowth.

### Induction of apoptosis in neuroblastoma and glioblastoma

Stimulation of TrkA with NGF leads to activation of prosurvival PI3K and ERK cascades and to proliferation, survival, and/or differentiation of neurons and majority of cancer cell lines^23^. However, paradoxically, TrkA acts as an inductor of apoptosis in neuroblastomas. TrkA expression in neuroblastomas correlates with patient survival, whereas TrkB overexpression is observed in aggressive neuroblastomas^24^. Overexpression of TrkA in SK-N-MC neuroblastoma and its derivative SH-SY5Y induces their apoptosis^25^. Moreover, the growing body of evidences indicates that TrkA decelerates growth of other brain tumors including glioblastomas^26^. TrkA overexpression in some glioma cell lines caused autophagy^26^.

It was found that a protein product of the cerebral cavernous malformation 2 (CCM2) gene causes cell death when co-expressed with TrkA^25^. CCM2 protein lacks any obvious enzymatic domains and is thought to function as a scaffolding or adaptor molecule able to selectively interact with a specific segment of the JM domain of TrkA, but not TrkB^27^. CCM2 is produced in neuroblastomas and glioblastomas, but not in other cancer cells.

**Fig. 7:**
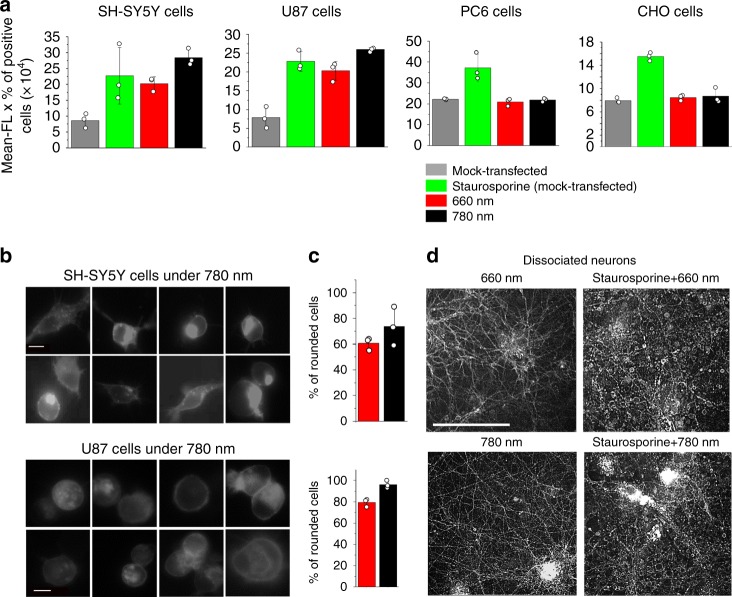
Regulation of apoptosis in neuroblastoma and glioblastoma by Dr-TrkA. **a** SH-S5Y5, U87, PC6-3, and CHO cells were co-transfected in 48-well plate with 250 ng mixture of the pCMVd2-mCherry-Dr-TrkA and pcDNA3.1 plasmids in the 1:1 mass ratio. The transfected cells were illuminated with either 660 nm or 780 nm light for 6 h, stained with annexin V, and analyzed using flow cytometry. As a positive control, cells transfected with pcDNA3.1 only (mock-transfected cells) were treated with 100 nM staurosporine. **b** Epifluorescence images of SH-SY5Y and U87 cells transfected with mCherry-Dr-TrkA illuminated with near-infrared (NIR) light, Scale bar, 10 μm. **c** Quantification of the rounded cells under either 660 nm or 780 nm light. Error bars represent s.d., *n* = 3 experiments. **d** Epifluorescence images of cultured rat cortical neurons transduced with adeno-associated virus serotype 9 (AAV9) encoding mCherry-Dr-TrkA. At 24 h after the transduction, neurons were illuminated for 6 h with either 660 nm or 780 nm light. As a positive control, the transduced neurons were treated with 100 nM staurosporine for 6 h. Scale bar, 100 μm. Error bars represent s.d., *n* = 3 experiments

Since Dr-TrkA contains the whole JM domain, we tested a possibility to use it for induction of apoptosis. We overexpressed Dr-TrkA in several cell types and analyzed apoptotic activity by annexin V staining and cell morphology (Fig. 7). The Dr-TrkA overexpression itself already initiated apoptosis in SH-SY5Y neuroblastoma and U87 glioblastoma, in contrast to PC6-3 pheochromocytoma and CHO epithelial cells (Fig. 7a). Triggering the Dr-TrkA kinase activity with NIR light further enhanced early apoptosis in SH-SY5Y and U87 cells up to 30%, as determined by annexin V staining, but did not cause notable apoptotic changes in PC6-3 and CHO cells (Fig. 7a). The late stages of apoptosis were also well detected by a large number of rounded SH-SY5Y and U87 cells (Fig. 7b, c). We concluded that the Dr-TrkA triggers apoptosis in tumor cells expressing CCM2 protein, acting similarly to that of TrkA.

### Light-induced activation of Dr-TrkA in neurons

To confirm a light-induced activation of Dr-TrkA in neurons we performed a cell-based enzyme-linked immunosorbent assay (ELISA) for ERK pathway. The 5 min 780 nm illumination was sufficient to observe an upregulation of the phosphorylated ERK in cultured rat cortical neurons and control HeLa cells (Supplementary Fig. 14). Further deactivation of the ERK pathway with 660 nm light was also fast in neurons, with a half-time of 5 min, but was substantially delayed (~45 min) in HeLa cells, likely reflecting slower dynamics of ERK signaling in the latter case.

To study the effect of Dr-TrkA activation in neurons, we transduced them with adeno-associated virus serotype 9 (AAV9) encoding mCherry-Dr-TrkA construct under CaMKII promoter. The Dr-TrkA expression in neurons did not cause their morphological changes and apoptosis under either 780 nm or 660 nm light (Fig. 7d). In contrast, addition of 100 nM of staurosporine caused cell rounding and death. Therefore, the light-dependent activation of Dr-TrkA was not toxic to neurons but toxic to neuroblastoma and glioma cells (Fig. 7a).

Together, these results suggest that Dr-TrkA could be used to selectively deplete tumor cells in the brain, leaving neurons intact.

### Spectral multiplexing with LOV2-based optogenetic tool

**Fig. 8:**
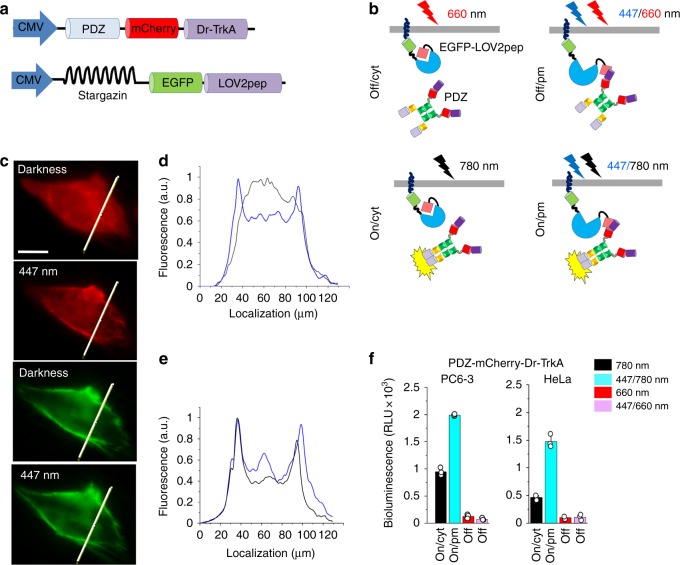
Regulation of Dr-TrkA activity with FR–NIR and localization with blue light. **a** Scheme of cytoplasmic PDZ-Cherry-TrkA and membrane-anchored stargazin-EGFP-LOV2pep constructs used for targeting of Dr-TrkA to the plasma membrane with blue light. **b** Translocation of the PDZ-mCherry-Dr-TrkA from the cytoplasm to the plasma membrane upon illumination with blue light and activation of the kinase with 780 nm light. **c** Top: Representative epifluorescence images of the HeLa cell in mCherry channel co-expressing PDZ-mCherry-TrkA and stargazin-EGFP-LOV2pep before and after 30 s of 447 nm illumination (0.3 mW cm^−2^). Bottom: Representative epifluorescence images of the same HeLa cell in EGFP channel. Scale bar, 10 μm. **d**, **e** The intensity profiles of the HeLa cells co-expressing PDZ-Cherry-TrkA and stargazin-EGFP-LOV2pep constructs kept in darkness (black line) or after 30 s of 447 nm light (0.3 mW cm^−2^) (blue line) imaged in mCherry channel (**d**) and EGFP channel (**e**). **f** Luciferase assay for Elk-1-dependent transcription in PC6-3 cells co-transfected with pPDZ-Cherry-Dr-TrkA, pStargazin-EGFP-LOV2pep, pFr-Luc, and pFA-Elk-1 plasmids. Cells were kept for 30 h under 780 nm light (0.3 mW cm^−2^), alternating 30 s pulses of 780 nm light (0.3 mW cm^−2^) and 447 nm light (0.3 mW cm^−2^), 660 nm light (0.3 mW cm^−2^) or alternating 30 s pulses of 660 nm light (0.3 mW cm^−2^) and 447 nm light (0.3 mW cm^−2^). After illumination cells were lysed and analyzed for luciferase activity. Error bars represent s.d., *n* = 3 experiments

As previously mentioned, several cytoplasmic proteins including products of oncogenes contain TrkA catalytic domain. These cytoplasmic kinases activate the same signaling pathways, as the TrkA membrane receptor, but to a lesser extent^13^. This fact and the observation that cyto-Dr-Trk variants activated MAPK/ERK pathway weaker than the myristoylated ones (Supplementary Fig. 9) prompted us to explore a light-induced recruitment of cyto-Dr-TrkA to the plasma membrane to enhance its control over cellular signaling. To achieve this without affecting Dr-TrkA kinase activity, the spectral range of light causing translocation from the cytoplasm to the plasma membrane should differ from the FR–NIR one. Therefore, we used blue-light-sensing properties of a LOV2 domain of *Avena sativa* phototropin 1 (AsLOV2) and designed a system, combining LOV2pep and PDZ constructs of Wagner and Glotzer^28^ and Dr-TrkA. Our system consisted of two components: a membrane-anchored stargazin-EGFP-LOV2pep protein and a cytoplasmic PDZ-mCherry-Dr-TrkA protein (Fig. 8a). In darkness, the peptide epitope (pep) is docked on the LOV2 domain, but upon illumination with blue light, it became exposed and bound to the engineered PDZ domain of erbin^28^, recruiting PDZ-mCherry-Dr-TrkA fusion to the plasma membrane (Fig. 8b). Cells expressing these two components demonstrated the cytoplasmic distribution of PDZ-mCherry-Dr-TrkA in darkness, which upon illumination with blue light translocated to the plasma membrane (Fig. 8c–e).

To access a synergetic effect of blue and NIR light on MAPK/ERK signaling, we co-transfected PC6-3 and HeLa cells with PDZ-mCherry-Dr-TrkA, stargazin-EGFP-LOV2pep, pFr-Luc, and pFA-Elk-1 constructs. Initially cells were kept under FR light to induce Dr-Trk-inactive state. Illumination of cells with 20 s pulse of 447 nm light, followed by 5 s pulse of 780 nm light, caused ~2.5-fold higher MAPK activation than in the cells exposed to 780 nm light only (Fig. 8f). Illumination with 447 nm light did not impact the level of Dr-TrkA inhibition by 660 nm light, indicating that recruitment of inactive Dr-TrkA to plasma membrane did not trigger activation of downstream signaling. In contrast, myristoylated Dr-TrkA activity remained nearly unaffected by illumination with blue light combined with either FR or NIR light (Supplementary Fig. 15a, b). Combination of constant blue and constant NIR light significantly activated MAPK/ERK signaling, whereas blue light alone did not (Supplementary Fig. 15c).

Thus, the combination of two spectrally independent light-sensing moieties, LOV2 and DrBphP-PCM, makes possible intracellular targeting of Dr-TrkA independently of its activation, enabling an additional spatial control over Trk signaling. The integrated Dr-Trk-LOV2 system acts as a two-stage regulator of the TrkA activity in which the first stage is triggered by NIR and the second by blue light.

### Activation of Dr-TrkA signaling in living mice

Since Dr-Trks are controlled with FR-NIR light in the so-called “tissue transparency window” (650–900 nm)^7,9^, they should be suitable for activation of downstream signaling in whole model mammals. Light in this spectral range is less phototoxic, scatters less, and penetrates tissues much deeper^8,9^, offering Dr-Trks an additional advantage over the common opto-kinases activatable with visible light.

**Fig. 9:**
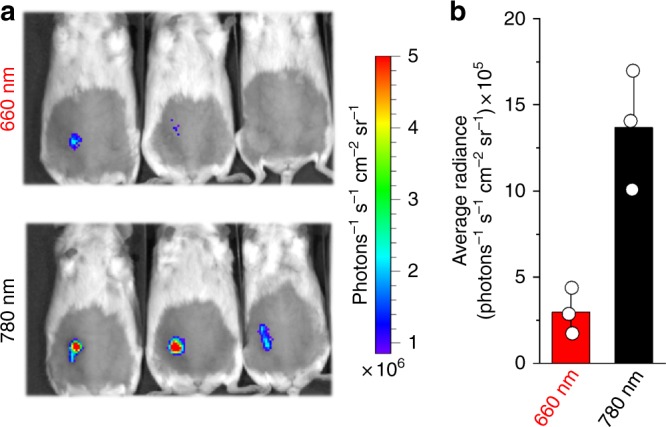
Light-induced activation of MAPK/ERK pathway in mice. **a** PC6-3 cells were co-transfected with pCMVd2-Dr-TrkA, pFA-Elk-1, and pFR-RLuc8 plasmids and injected 25 h later in mammary glands of female mice. Mice were kept in transparent cages and illuminated from below with either 660/25 nm or 780/25 nm LED arrays at 3 mW cm^−2^ for 19 h. Then, animals were injected with a coelenterazine substrate for RLuc8 luciferase through retro-orbital vein and immediately imaged with an IVIS Spectrum instrument. **b** Quantification of the RLuc8 reporter signals shown in **a**. Error bars represent s.d., *n* = 3 experiments

For a proof of principle, we tested Dr-TrkA ability to light-activate MAPK/ERK pathway in vivo using the reporting system shown in Fig. 1c. For this, we injected PC6-3 cells co-transfected with Dr-TrkA and downstream Elk-1 and RLuc8 luciferase reporters in mammary glands of mice (~1–2 mm deep). Mice spent overnight under 780 nm light had up to 4-fold higher luciferase expression than the mice kept under 660 nm light during the same time (Fig. 9).

These results demonstrate that Dr-Trks enable non-invasive activation of downstream signaling in live animals.

## Discussion

We have developed the FR–NIR light-controlled Dr-TrkA and Dr-TrkB kinases from DrBphP-PCM and cytoplasmic domains of TrkA and TrkB. Dr-Trks are active under 780 nm NIR light, whereas illumination with 660 nm FR light inactivates them. We substantially increased the photodynamic range of the initial DrBphP-PCM-cyto-Trk fusions by inserting rigid linkers of four α-helical repeats between DrBphP-PCM and cyto-Trk domains. The final Dr-TrkA and Dr-TrkB exhibit up to 33-fold activation range of the downstream signaling (Fig. 2).

In contrast to chemical inhibitors, which simultaneously target multiple kinases, Dr-Trks permit to achieve specific inhibition of the corresponding pathway with FR light. In this regard, Dr-Trk opto-kinases are as selective as “bump-and-hole” approach, which specifically inhibits a single mutated protein kinase (called “analog kinase”) without affecting the activity of its endogenous analogs^29^.

Opto-kinases engineered from LOV^13^, CRY^3^, and CBD^29^ can be activated with blue (LOV, CRY) or green light (CBDs) and inactivated in darkness. In contrast, Dr-Trks are activated by NIR and inhibited by FR light. This property make them similar to opto-kinases developed from pdDronpa^6^. However, the latter ones require violet and cyan light, which is phototoxic and overlays with activation spectra of common optogenetic tools.

It has been shown that a two-component FR–NIR optogenetic system based on the heterodimerization of a RpBphP1 bacterial phytochrome with its binding partner RpPpsR2 is spectrally compatible with LOV-based optogenetic tools, enabling simultaneous regulation of several processes in a cell^30,31^. Our current results indicate that the FR–NIR light sensitivity of the DrBphP-based opto-kinases also allows their crosstalk-free combination with the visible-light optogenetic tools and fluorescent proteins.

Moreover, as far as Dr-Trks are switchable with the FR–NIR light, they are applicable to the regulation of cell signaling in vivo, as presented in Fig. 9.

To date, an LAPD (light-activated phosphodiesterase)^32^ and an IlaC adenylate cyclase^33^ were the only optogenetically controlled with FR–NIR light enzymes engineered from bacterial phytochromes. LAPD was created by fusing catalytic domain of human phosphodiesterase 2A to DrBphP-PCM, and IlaC was designed by fusion of a BphPG bacterial phytochrome to an adenylate cyclase domain from *Nostoc sp*. bacteria. Both these tools exploit the transduction of an absorbed light signal from the PCM to the C-terminal output enzyme through the native helical linkers, resulting in the conformational changes of the enzymatic domain, modulating its activity. In contrast, Dr-Trks have artificially introduced rigid linkers, which substantially elongate the native helices, allowing the kinase domains in the DrBphP-PCM dimer to reversibly monomerize–dimerize, resulting in their inactivation and activation, respectively.

The Dr-Trk experimental validation suggests that the Dr-Trk modular engineering approach could be applied to kinase domains of homologous RTKs to regulate their signaling with light. Moreover, we anticipate that this engineering approach could also be employed to develop other light-controllable enzymes and transcription factors whose activity depends on the monomerization–dimerization transition.

## Methods

### Molecular cloning

The plasmid encoding the full-length DrBphP was kindly provided by J. Ihalainen. The pFr-Luc reporter and pFA-Elk-1 transactivator plasmids were from Stratagene. The plasmid encoding PH-Akt-GFP reporter was from Addgene (#51465). The plasmid pKA-121-Myr-mCherry-3xSAG-RpBphP1 has been described earlier^34^. Rat TrkB sequence was amplified from the plasmid pEGFP-N1-TrkB (Addgene #32500), and rat TrkA was amplified from rat brain complementary DNA (cDNA) library. Mammalian expression vectors were constructed by replacement of RpBphP1 sequence in a pKA-121-Myr-mCherry-3xSAG-RpBphP1 vector^34^ with either Dr-TrkA or Dr-TrkB using *Age*I/*Xho*I sites. The resulting myr-mCherry-3xSAG-Dr-Trks were cloned into a pFN23-HaloTag-CMVd2-Flexi vector (Promega) vector at *Hin*dIII/*Xho*I sites. A new *Xho*I site was inserted, resulting in the vector termed pCMVd2. A mCherry tag was removed, and α-helical linkers of various length were added between the DrBphP-PCM and cyto-Trk moieties. The resulting plasmids encoded myristoylated DrBphP-PCM-linker-cyto-Trk protein variants under the CMVd2 promoter. To construct a bicistronic vector for detection of PIK3 pathway activation, PH-Akt-EGFP was amplified from Addgene plasmid (#51465) and inserted into a pcDNA3.1+ using *Nhe*I/*Eco*RI sites. An IRES2 and myr-mCherry-3xSAG-Dr-TrkA sequences were inserted after PH-Akt-EGFP one using *Eco*RI/*Bam*HI sites. The resulting bicistronic plasmid encoded PH-Akt-EGFP-IRES2-Dr-TrkA under CMV promoter. To construct a pPDZ-Dr-TrkA vector for spectral multiplexing, a 2xPDZ-domain from the Addgene (#80407) was amplified and cloned upstream the mCherry-Dr-TrkA plasmid in the pcDNA3.1+ plasmid, using sites *Hin*dIII, *Bam*HI, and *Xho*I, respectively. The major plasmids designed in this study are listed in Supplementary Table 1. The protein sequences of the Dr-TrkA, Dr-TrkB, cyto-Dr-TrkA, cyto-Dr-TrkB, DrBphP-PCM-cyto-TrkB, and DrBphP-PCM-DHp-cyto-TrkB fusions are presented in Supplementary Table 2.

### Mammalian cell culture and transfection

PC6-3 cells and SH-SY5Y cell lines were a gift of Dr. Lindholm (University of Helsinki, Finland). PC6-3 were cultured in RPMI-1640 medium supplemented with 10% horse serum (HS) and 5% fetal bovine serum (FBS) (both from Biowest). SH-SY5Y cells were cultured in Dulbecco's Modified Eagle's medium (DMEM) supplemented with 10% FBS (Biowest). HeLa, NIH3T3, and U87 cell lines were obtained from ATCC and cultured in DMEM supplemented with 10% FBS (Biowest). No additional authentication of the cell lines was performed. U87 cell line (HTB-14 in ATCC) is considered misidentified because its genetic profile differs from that of the original tumor obtained in 1968; nevertheless, the ATCC-derived U87 is considered to be the human glioblastoma of unknown origin^35^. For live cell microscopy, cells were cultured on the poly-l-lysine (M-Specialty Media)-coated coverslips (5 μg ml^−1^). For screening of Dr-Trk fusions, 20,000 of PC6-3 cells were seeded in 0.5 ml medium per well in 24-well plates and transfected with 1 µg of pCMVd2-Dr-Trk, pFr-Luc, and pFA-Elk-1 plasmid mixture (mass ratio 1:100:5). Prior to transfection, a Turbofect reagent (Thermo Fisher Scientific) was added to plasmid DNA at a volume-to-mass ratio of 2:1. After 6 h of incubation, the transfection medium was changed to a serum-starving medium (RPMI with 1% HS and 25 µM BV). Cells then were kept for 30 h under either 660 nm (0.5 mW cm^−2^) or 780 nm (0.5 mW cm^−2^) light. For analysis of Elk-1-, CREB-, and c-Jun-dependent transcription activation, PC6-3, HeLa, SH-SY5Y, and NIH3T cells were seeded on 24-well plates and transfected as above but with different plasmid ratio (1:100:1). After transfection, the medium was changed to RPMI with 1% HS for PC6-3, DMEM with the 1% of FBS for HeLa, and DMEM with 0.1% FBS for SH-SY5Y and NIH3T3 cells.

### Bioluminescence assay

After removal of culture medium, cells were frozen at −80 °C to ensure efficient cell disruption. Cells were lysed in 100 µl of lysis buffer (20 mM Tris-HCl, 10% glycerol, 0.1% Triton X-100, 1 mM PMSF, 0.1% β-mercaptoethanol, pH 8.0) for 30 min at room temperature on swinging platform shaker. Luciferase assay was performed in 96-well half-area white plates (Costar) by mixing 10 μl of cell lysate with 20 μl of firefly luciferase substrate (Nanolight Technology). Bioluminescence was measured immediately with a Victor X3 multilabel plate reader (Perkin Elmer), and data were analyzed with an OriginPro 8.6 software.

### Immunoblotting

For immunoblotting, HeLa cells were plated in 6-well plates and transfected at 90% confluence with linear polyethylenimine (PEI)/DNA complexes. Transfection mixtures were prepared by dilution of 4 µg DNA with 400 µl DMEM medium and 8 μl PEI (1 mg ml^−1^, PolyScience, #23966-1). For detection of ERK phosphorylation, a pcDNA-mCherry-Dr-TrkA plasmid was mixed with a pcDNA3.1+ at mass ratio 1:20. For detection of TrkA phosphorylation, this ratio was 1:5. PEI/DNA complexes were formed for 15 min and added to the wells drop-wise. After 6 h, the medium was changed to DMEM with 0.5% FBS and 25 µM BV. Then, cells were kept under 660 nm light for additional 20 h. Dr-TrkA was activated for 5 min by transferring plates under 780 nm light. After that cells were put on ice, washed once with ice-cold phosphate-buffered saline (PBS), and lysed in 300 µl of ice-cold RIPA buffer (Thermo Fisher Scientific, #89900) supplemented with phosphatase (Thermo Fisher Scientific, #88265) and protease (Thermo Fisher Scientific, #A32953) inhibitors. Cell lysis was performed for 5 min. Lysates were centrifuged at 12,000 rpm for 20 min in an Eppendorf centrifuge at 4 °C. For phospho-ERK detection, 20 µl of lysate per lane was loaded to 10% gel. For phospho-Trk detection, 30 μl of lysate per lane was loaded to 7.5% gel. Proteins were separated by sodium dodecyl sulfate–polyacrylamide gel electrophoresis (SDS-PAGE) and transferred to nitrocellulose membranes. Membranes were blocked in 5% solution of non-fat dry milk for 2 h and were incubated overnight at 4 °C with monoclonal antibodies against phosphorylated ERK (Cell Signaling, #9101) diluted 1:2000 or phosphorylated TrkA (Cell Signaling, #9141) diluted 1:1000. For total ERK and total Trk detection, rabbit anti-Erk1/2 monoclonal antibodies (mAbs; Cell Signaling, #4695) diluted 1:1000 and rabbit anti-TrkA mAbs (Cell Signaling, #30697) diluted 1:1000 were used. For glyceraldehyde 3-phosphate dehydrogenase (GAPDH) detection, mouse anti-GAPDH mAbs (Santa Cruz, sc-32333) were used in 1:1000 dilution. Then, the membranes were washed 4 times by Tris-buffered saline (TBS) with 0.5% Tween and incubated with goat anti-rabbit IgG-horseradish peroxidase (HRP) (Cell Signaling, #7074) diluted 1:3000 or goat anti-mouse IgG-HRP (Santa Cruz, sc-2005) diluted 1:1000 for 2 h and washed 5 times again. For the dilution of all antibodies on all incubation steps, 5% non-fat dry milk solution in TBS was used. TBS with 0.05% Tween was used in all wash steps. Bioluminescence was detected using a Clarity Western ECL Substrate (Bio-Rad). Images were taken with a ChemiDoc Imaging system.

### Cell viability assay

PC6-3 cells were seeded at a density 80,000 cells per well into 24-well plates in RPMI-1640 medium supplemented with 10% HS and 5% FBS. The next day, the medium was changed to serum-starving medium (RPMI with 1% HS and 25 µM BV) supplemented with 100 nM of inhibitors. Cells were grown for additional 30 h, washed with PBS, detached with trypsin/EDTA (Gibco), collected, and re-suspended in 0.2 ml of PBS. Cells were then stained by addition of 2 µl propidium iodide solution (1 mg ml^−1^). Cells were immediately analyzed using FACS Accuri (BD Biosciences). Viable cells were calculated for each sample by subtraction of a number of propidium iodide-positive cells (dead cells) from the total number of cells.

### Inhibition of Elk-1 and CREB-dependent transcription

Chemical inhibitors of Trk activity, such as entrectinib (Trk family inhibitor), BMS-754807 (inhibitor of insulin receptor IGF1R), and AZ23 (Trk family inhibitor) were kindly provided by a High-Throughput Screening unit of FIMM, University of Helsinki. PC6-3 cells were plated and transfected as before. After transfection, the medium was changed to serum-starving medium (RPMI with 1% HS and 25 µM BV) and supplemented with 100 nM of inhibitors. Cells were grown under 660 nm or 780 nm illumination for 30 h before bioluminescence assay.

### Activation of PI3K signaling by Dr-Trks

PC6-3 cells were plated on poly-l-lysine-coated glass-bottom 35 mm dishes (Nunc) and transfected with 3 μg of pPH-Akt-EGFP-IRES2-Dr-TrkA and pFR-Luc plasmid mixture (1:1 mass ratio) using Lipofectamine 3000. The pFR-Luc plasmid was used as carrier DNA here to reduce expression of PH-Akt-EGFP. The medium was changed to RPMI with 1% HS and 25 µM BV and cells were grown for 24 h under 660 nm illumination (0.5 mW cm^−2^). At 4 h before imaging, the medium was changed to serum-free DMEM to avoid non-specific activation of the PI3K pathway. Cells were imaged in a cell imaging medium (Life Technologies). During imaging, cells were kept at 37 °C under 660 nm illumination. To induce PH-Akt-EGFP translocation to the plasma membrane, 780 nm light for 3 min was applied, and images were taken every 2 min. To induce dissociation of PH-Akt-EGFP from the plasma membrane to cytosol, the cells were illuminated with 660 nm light in intervals between taking images. To test reversibility of Dr-TrkA activity, cells were repeatedly illuminated with 780 nm light for 10 min followed by 660 nm light, and images were taken every 20 s. Data were analyzed using a SlideBook v. 6.0.8 and an ImageJ v. 1.50b software.

### Activation of calcium signaling by Dr-Trks

For optogenetic activation, HeLa cells were plated in 35 mm glass-bottom dishes covered with poly-l-lysine and co-transfected with 1.25 µg of mCherry-Dr-TrkA and 1.25 µg GCaMP6m (Addgene #40754) plasmids using Lipofectamine 3000 (Thermo Fisher Scientific). To study activation with NGF (a positive control), HeLa cells were co-transfected with 1.25 µg of TrkA-DsRed2 (Addgene #24093) and 1.25 µg GCaMP6m plasmids. At 6 h after transfection, medium was changed to DMEM with 2.5% FBS and 25 mM BV. The medium was changed 24 h later to serum-free DMEM and cells were starved for 6 h under 660 nm illumination. Then, the cells were washed 3 times with calcium-free Hank’s balanced salt solution (HBSS), and HBSS with calcium (Thermo Fisher Scientific) was added. In the positive control, HeLa cells were perfused with calcium-containing HBSS with or without K252a and induced with 50 nM of NGF.

To light-activate Dr-Trk in HeLa cells, 660 nm and 780 nm LED arrays were positioned over the cell culture Petri dish at ~45° each. The cells were initially illuminated with 660 nm light; after 5 s of imaging, 780 nm light was applied for 20 s, while 660 nm light was switched off; and after that 660 nm light was switched on again. Epifluorescence images of HeLa cells with GCaMP6m were taken each 1 s using SlideBook v. 5.0 software (Intelligent Imaging Innovations). The image stacks were then processed by first subtracting the background autofluorescence for the entire image stack using the SlideBook software. Next, the obtained images were exported to an ImageJ v. 1.52 software (National Institutes of Health) and were first pixel-by-pixel aligned using the TurboReg plugin. Then, the image stacks for cells illuminated with 660 nm light only, for cells illuminated with 660 nm and 20 s of 780 nm light, illuminated with 660 nm light and treated with NGF, and illuminated with 660 nm light and treated with K252a inhibitor were analyzed separately using the ImageJ software. Specifically, for time-dependent changes in a relative fluorescence intensity, the circular regions of interest were placed at the cytosolic regions and quantified for the whole image stacks for each of the aforementioned condition. Next, the relative fluorescence intensity was calculated for each dataset, and the resultant datasets were normalized to the respective maximal values using an OriginPro v. 2018 software (OriginLab). A half-time of calcium increase was determined as a time at which the normalized relative fluorescence intensity of GCAMP6m increased to the half-maximal value after the application of a stimulus (780 nm light or growth factor). A half-time of calcium decrease was determined as a time at which the normalized relative fluorescence intensity of GCAMP6m decreased to the half-maximal value after the peak.

### Neurite outgrowth assay

PC6-3 cells were seeded at 15,000 cells per well on poly-l-lysine-coated coverslips and transfected with 1 μg of either pEGFP-IRES2-Dr-TrkA or pEGFP-IRES2-Dr-TrkB plasmids. The medium was changed to RPMI supplemented with 1% HS and 25 µM BV, and cells were grown for 72 h under either 780 nm or 660 nm light (both of 0.3 mW cm^−2^). After that cells were stained with a Dil stain kit, according to the protocol provided by the supplier (Thermo Fisher Scientific). Then, cells were washed with PBS, fixed with 3% paraformaldehyde, and mounted with UltraCruz mounting medium (Santa Cruz Biotechnology). A cell was considered as one bearing neurite if it had at least one neurite longer than a diameter of its body. Only green cells were counted from three different coverslips. Cell imaging was performed using an Olympus IX83 inverted epifluorescence microscope equipped with a 200 W metal-halide arc lamp (Lumen 220Pro, Prior), a 60× 1.35NA oil immersion objective lens (UPlanSApo, Olympus), and standard sets of filters (Chroma).

### Spectral multiplexing

For experiments with blue-light-activatable LOV2 domain, PC6-3 and HeLa cells were plated as above. Transfection of the cells was performed with pcDNA-PDZ-Cherry-Dr-TrkA, pStargazin-LOV2pep, pFr-Luc, and pFA-Elk-1 plasmids at a 3:1:20:1 ratio. Recruiting of Dr-TrkA to the plasma membrane was achieved by 20 s pulses of 447 nm light (0.3 mW cm^−2^) followed by 5 s pulses of 780 mn light (0.3 mW cm^−2^). To test spectral responses of Dr-Trks, continuous 447 nm light (0.3 mW cm^−2^), 780 nm light (0.3 mW cm^−2^), and 660 nm light (0.3 mW cm^−2^) was used.

### Assay for Dr-Trk stability

HeLa cells were transfected with mCherry-Dr-TrkA plasmid using Lipofectamine 3000 in 24-well plates (1 µg of plasmid per well) and kept whether under 660 nm or 780 nm light. Cells were lysed in 100 µl of PBS with 1% Tween-20, and mCherry fluorescence of lysates was measured in 96-well glass-bottom plates using the Victor X3 plate reader (Perkin Elmer). For analysis of Dr-Trk stability using western blot, HeLa cells plated in 6-well plates were transfected with 4 μg of either Dr-TrkA or mCherryDr-TrkA plasmids per well using PEI.

### Annexin V cell staining

For annexin V staining, SH-SY5Y cells and U87 cells were seeded in 48-well plates and co-transfected with 250 ng mixture of the pCMVd2-mCherry-Dr-TrkA and pcDNA3.1+ plasmids in the 1:1 mass ratio using Lipofectamine 2000. After 6 h, the medium was changed to the medium with 25 μM BV and cells were grown for 18 h under 660 nm light. The transfected cells were stimulated with either 660 nm or 780 nm light for 6 h. Then, cells were collected by centrifugation, stained with annexin V AlexaFluor 488 Ready-Flow reagent, and immediately analyzed using flow cytometry.

### Production of AAV

AAV9 was produced in an AAV Gene Transfer facility of Medicum, University of Helsinki. For the AAV9 production, a pAAV-CW3SL-Myr-Cherry-Dr-TrkA plasmid, based on the pAAV-CW3SL-EGFP plasmid (Addgene #61463), was used.

### Experiments with dissociated neurons

Rat cortical neurons were prepared by a Neuronal Cell Culture unit of University of Helsinki from late embryonic stage (E17–18) rat embryos. Animal work was performed in accordance with the ethical guidelines of the European Convention and regulations of the Ethics Committee for Animal Research of University of Helsinki. Neurons were plated in a 96-well white glass-bottom plate (Corning) at a plating density of 20,000 cells per well, grown for 7 days in vitro, and transduced with an AAV9 encoding mCherry-Dr-TrkA under CAMKII promoter for 24 h. After that, the transduction medium was changed to a medium with 25 μM of BV. For detection of apoptosis, plates with neurons were kept under either 660 nm or 780 nm light for 6 h. As a control for apoptosis, neurons were treated with 100 nM staurosporine. Imaging of neurons was performed using an Opera Phenix High-Content microscope (Perkin Elmer) equipped with a 60× water-immersion objective.

### Cell-based ELISA

For cell-based ELISA, isolated neurons and HeLa cells were plated to 96-well microplates at 20,000 cells per well. Neurons were transduced with the AAV9 (based on pCAMKII-mCherry-Dr-TrkA plasmid), and HeLa cells were transfected with the pcDNA-Dr-TrkA and pcDNA3.1+ plasmid mixture at mass ratio 1:10 using Lipofectamine 3000 (Thermo Fisher Scientific). For detection of ERK phosphorylation, pcDNA-mCherry-Dr-TrkA plasmid was mixed with pcDNA3.1+ plasmid at mass ratio 1:20. For Hela cells, the medium was changed 6 h after transfection to DMEM with 0.5% FBS and 25 µM BV. For neurons, a half of the medium was changed to the Neurobasal Medium without phenol red, supplemented with 25 µM BV, GlutaMAX, and B-27 Supplement serum free (all from Thermo Fisher Scientific) 24 h after transduction. Then, the cells were kept under 660 nm light for 20 h. Dr-TrkA was activated by transferring the plates with cells under 780 nm light for 5 min. After that, the plates were illuminated with 660 nm light for 5, 10, 30, 60, and 90 min. At each time point, the plates with stimulated cells were transferred to ice and fixed with 8% paraformaldehyde for 15 min. After fixation, the cells were permeabilized for 30 min with 0.05% Triton and blocked with blocking solution (5% non-fat dry milk in TBS with 0.01% Tween) for 2 h. After blocking, the plates were incubated overnight with anti-phospho-ERK antibodies (Cell Signaling Technology) in 1:250 dilution. Next morning, the plates were washed by TBS with 0.5% Tween and incubated with goat anti-rabbit HRP conjugate (1:5000) for 2 h at room temperature. The color development was detected using a 1-Step Ultra TMB-ELISA substrate solution (Thermo Fisher Scientific). Absorbance was measured at 450 nm using a Victor X3 plate reader (Perkin Elmer) and analyzed with an OriginPro v.8.6 software.

### Light activation and imaging in mice

To study light-induced activation of MAPK/ERK pathway by Dr-TrkA, PC6-3 cells were injected subcutaneously in mice. PC6-3 cells were co-transfected with the pCMVd2-Dr-TrkA, pFA-Elk-1, and pFR-RLuc8 plasmids in the 1:10:90 mass ratio. Medium was changed 6 h after the transfection to a RPMI-1640 medium, supplemented with 10% HS, 5% FBS, 2 mM l-glutamine, and 25 µM BV and cells were incubated overnight before the injection. For injection, Swiss Webster 2- to 3-month-old female mice (National Cancer Institute, NIH) with body weights of 22–25 g were used. The 3 × 10^6^ of PC6-3 cells were re-suspended in 100 μl of RPMI-1640 medium with 2 mM l-glutamine and injected subcutaneously in the mammary glands of mice 25 h after the transfection. After injection, mice were placed in transparent cages and illuminated from the bottom with either a 660/25 nm LED array (3 mW cm^−2^) or a 780/25 nm LED array (3 mW cm^−2^) for 19 h. For imaging, fur on the bellies of the mice was removed using a depilatory cream. Each experimental group contained 3 mice. For bioluminescence detection, the animals were imaged with an IVIS Spectrum instrument (Perkin Elmer). Bioluminescence was detected with an open emission filter. Throughout imaging, the animals were maintained under anesthesia with 1.5% vaporized isofluorane. Prior to imaging, 80 μg of Inject-A-Lume coelenterazine substrate (NanoLight Technology) for RLuc8 luciferase was intravenously injected through a retro-orbital vein. Images were analyzed using Living Image 3.0 software (Perkin Elmer). All animal experiments were performed in an AAALAC (Association for Assessment and Accreditation of Laboratory Animal Care International)-approved facility using protocols approved by the Albert Einstein College of Medicine Animal Usage Committee. A total of 20 mice were used in this study.

## Supplementary information

Supplementary Information

Reporting Summary

Flow Cytometry Reporting Summary

Source Data

## Data Availability

The main data supporting the findings of this study are available in the article and its Supplementary Information. The source data underlying all Figures and Supplementary Figures are provided as a Source Data file. The additional data are available from the corresponding author on reasonable request.
