# Supplementary material for: Neurotrophin receptor tyrosine kinases regulated with near-infrared light
Source: Nat Commun. 2019 Mar 8;10:1129. doi: 10.1038/s41467-019-08988-3 (PMC6408446; doi:10.1038/s41467-019-08988-3)
Supplement: Supplementary file 1 — Supplementary Information [file 41467_2019_8988_MOESM1_ESM.pdf]

Neurotrophin receptor tyrosine kinases regulated with near-infrared light

Anna V. Leopold, Konstantin G. Chernov, Anton A. Shemetov and Vladislav V. Verkhusha

Supplementary Information

Supplementary Table 1. List of major plasmids designed in this study.

Plasmid	Vector backbone	Promoter	Insert
pCMVd2-DrBphP-PCM-TrkB	pCMVd2	CMVd2	*HindIII*-Myr-3xSAG-Dr-*KpnI*-TrkB-XhoI
pCMVd2-Dr-hel1-TrkB	pCMVd2	CMVd2	*HindIII*-Myr-3xSAG-Dr-hel1-TrkB-XhoI
pCMVd2-Dr-hel2-TrkB	pCMVd2	CMVd2	*HindIII*-Myr-3xSAG-Dr-hel2-TrkB-XhoI
pCMVd2-Dr-hel3-TrkB	pCMVd2	CMVd2	*HindIII*-Myr-3xSAG-Dr-hel3-TrkB-XhoI
pCMVd2-Dr-hel4-TrkB	pCMVd2	CMVd2	*HindIII*-Myr-3xSAG-hel4-TrkB-XhoI
pCMVd2-Dr-hel5-TrkB	pCMVd2	CMVd2	*HindIII*-Myr-3xSAG-hel5-TrkB-XhoI
pCMVd2-Dr-DHp-TrkB	pCMVd2	CMVd2	*HindIII*-Myr-3xSAG-Dr-M614-TrkB-XhoI
pCMVd2-Dr-hel1-TrkA	pCMVd2	CMVd2	*HindIII*-Myr-3xSAG-Dr-hel1-TrkB-XhoI
pCMVd2-Dr-hel2-TrkA	pCMVd2	CMVd2	*HindIII*-Myr-3xSAG-Dr-hel2-TrkB-XhoI
pCMVd2-Dr-hel3-TrkA	pCMVd2	CMVd2	*HindIII*-Myr-3xSAG-Dr-hel3-TrkB-XhoI
pCMVd2-Dr-hel4-TrkA	pCMVd2	CMVd2	*HindIII*-Myr-3xSAG-Dr-hel4-TrkB-XhoI
pCMVd2-Dr-hel5-TrkA	pCMVd2	CMVd2	*HindIII*-Myr-3xSAG-Dr-hel5-TrkB-XhoI
pcDNA3.1+-EGFP-PH-Akt-IRES2-TrkA	pcDNA3.1+	CMV	*NheI*-PH-Akt-BamHI-EGFP-EcoRI-IRES2-*SalI*-Myr-3xSAG -Dr-hel4-TrkA-*XhoI*
pcDNA3.1+-EGFP-PH-Akt	pcDNA3.1+	CMV	*NheI*-PH-Akt-BamHI-EGFP-*EcoRI*
pEGFP-IRES2-Dr-TrkA	pEGFP-C1	CMV	*EcoRI*-IRES2-*SalI*-Myr-3xSAG Dr-hel4-TrkA-*XhoI*
pEGFP-IRES2-Dr-TrkB	pEGFP-C1	CMV	*EcoRI*-IRES2-*SalI*-Myr-3xSAG -Dr-hel4-TrkA-*XhoI*
pKA-Dr-hel4-TrkA	pKA	CMV	*AgeI*-Dr-hel4-TrkA-*XhoI*
pKA-Dr-hel4-TrkB	pKA	CMV	*AgeI*-Dr-hel4-TrkB-*XhoI*
cyto-Dr-TrkA	pCMVd2	CMVd2	pCMVd2-*HindIII*-Dr-hel4-TrkA-*XhoI*
cyto-Dr-TrkB	pCMVd2	CMVd2	pCMVd2-*HindIII*-Dr-hel4-TrkB-*XhoI*
pcDNA3.1+-PDZ-mCherry-Dr-TrkA	pcDNA3.1+	CMV	*HindIII*-PDZ-mCherry-3xSAG-Dr-hel-4-TrkA-*BamHI*
pcDNA3.1+-PDZ-Dr-TrkA	pcDNA3.1+	CMV	HindIII-PDZ-3xSAG-Dr-hel4-TrkA-BamHI
pcDNA3.1+-Myr-Cherry-Dr-TrkA	pcDNA3.1+	CMV	*HindIII*-Myr-mCherry-3xSAG-Dr-hel-4-TrkA-*BamHI*
pcDNA3.1+-mCherry-Dr-TrkA	pcDNA3.1+	CMV	*HindIII*-mCherry-3xSAG-Dr-hel-4-TrkA-*BamHI*
pcDNA3.1+Myr-Cherry-Dr-TrkB	pcDNA3.1+	CMV	*HindIII*-Myr-mCherry-3xSAG-Dr-hel4-TrkB-*XhoI*
pAAV-CW3SL-Myr-Cherry-Dr-TrkA	pAAV-CW3SL	CaMKII	*HindIII*-Myr-mCherry-Dr-TrkA-*XhoI*

Supplementary Table 2. Protein sequences of major proteins designed in this study.

Dr-TrkA	MGCIKSKRKDALYKEFSAGSAGSAGMSRDPLPFFPPLYLGGPEI<br>TTENCEREPIHIPGSIQPHGALLTADGHSGEVLQMSLNAATFLG<br>QEPTVLRGQTLAALLPEQWPALQAALPPGCPDALQYRATLDW<br>PAAGHLSLTVHRVGELLILEFEPTEAWDSTGPHALRNAMFALE<br>SAPNLRALAEVATQTVRELTGFDRVMLYKFAPDATGEVIAEAR<br>REGLHAFLGHRFPASDIPAQARALYTRHLLRLTADTRAAAVPL<br>DPVLNPQTNAPTPLGGAVLRATSPMHMQYLRNMGVGSSLSVS<br>VVVGGQLWGLIACHHQTPYVLPPDLRTTLEYLGRLLSLQVQVK<br>EAADVAAFRQSLREHHARVALAAAHSLSPHDTLSDPALDLLGL<br>MRAGGLILRFEGRWQTLGEVPPAPAVDALLAWLETQPGALVQ<br>TDALGQLWPAGADLAPSAAGLLAISVGEGWSECLVWLRPELR<br>LEVAWGGATPDQAKDDLGPRHSFDTYLEEKRGYAEPWHPGEI<br>EEAQDLRDTLTGALGEAEAAAKEAAAKEAAAKEAAAKANKC<br>GQRSKFGINRPAVLAPEDGLAMSLHFMTLGGSSLSPTEGKGSG<br>LQGHIMENPQYFSDTCVHHIKRQDIILKWELGEGAFGKVFLAEC<br>YNLLNDQDKMLVAVKALKETSENARQDFHREAELLTMLQHQ<br>HIVRFFGVCTEGGPLLMVFEYMRHGDLNRFLRSHGPDAKLLAG<br>GEDVAPGPLGLGQLLAVASQVAAGMVYLASLHFVHRDLATRN<br>CLVGQGLVVKIGDFGMSRDIYSTDYYRVGGRTMLPIRWMPPES<br>ILYRKFSTESDVWSFGVVLWEIFTYGKQPWYQLSNTEAIECITQ<br>GRELERPRACPPDVYAIMRGCWQREPQQRLSMKDVHARLQAL<br>AQAPPSYLDVLG
Dr-TrkB	MGCIKSKRKDALYKEFSAGSAGSAGMSRDPLPFFPPLYLGGPEI<br>TTENCEREPIHIPGSIQPHGALLTADGHSGEVLQMSLNAATFLG<br>QEPTVLRGQTLAALLPEQWPALQAALPPGCPDALQYRATLDW<br>PAAGHLSLTVHRVGELLILEFEPTEAWDSTGPHALRNAMFALE<br>SAPNLRALAEVATQTVRELTGFDRVMLYKFAPDATGEVIAEAR<br>REGLHAFLGHRFPASDIPAQARALYTRHLLRLTADTRAAAVPL<br>DPVLNPQTNAPTPLGGAVLRATSPMHMQYLRNMGVGSSLSVS<br>VVVGGQLWGLIACHHQTPYVLPPDLRTTLEYLGRLLSLQVQVK<br>EAADVAAFRQSLREHHARVALAAAHSLSPHDTLSDPALDLLGL<br>MRAGGLILRFEGRWQTLGEVPPAPAVDALLAWLETQPGALVQ<br>TDALGQLWPAGADLAPSAAGLLAISVGEGWSECLVWLRPELR<br>LEVAWGGATPDQAKDDLGPRHSFDTYLEEKRGYAEPWHPGEI<br>EEAQDLRDTLTGALGEAEAAAKEAAAKEAAAKEAAAKAKLA<br>RHSKFGMKGPASVISNDDDSASPLHHISNGSNTPSSSEGGPDAVI<br>IGMTKIPVIENPQYFGITNSQLKPDTFVQHIKRHNIVLKRELGEG<br>AFGKVFLAECYNLCPEQDKILVAVKTLKDASDNARKDFHREAE<br>LLTNLQHEHIVKFYGVCVEGDPLIMVFEYMKHGDLNKFLRAH<br>GPDAVLMAEGNPPTELTQSQMLHIAQQIAAGMVYLASQHFVH<br>RDLATRNCLVGENLLVKIGDFGMSRDVYSTDYYRVGGHTMLP<br>IRWMPPESIMYRKFTTESDVWSLGVVLWEIFTYGKQPWYQLSN

	NEVIECITQGRVLQRPRTCPQEVYELMLGCWQREPHTRKNIKNI<br>HTLLQNLAKASPVYLDILG
cyto-Dr-TrkA	MSRDPLPFFPPLYLGGPEITTENCEREPIHIPGSIQPHGALLTADG<br>HSGEVLQMSLNAATFLGQEPTVLRGQTLAALLPEQWPALQAA<br>LPPGCPDALQYRATLDWPAAGHLSLTVHRVGELLILEFEPTEA<br>WDSTGPHALRNAMFALESAPNLRALAEVATQTVRELTGFDRV<br>MLYKFAPDATGEVIAEARREGLHAFLGHRFPASDIPAQARALY<br>TRHLLRLTADTRAAAVPLDPVLNPQTNAPTPLGGAVLRATSPM<br>HMQYLRNMGVGSSLSVSVVVGGQLWGLIACHHQTPYVLPPDL<br>RTTLEYLGRLLSLQVQVKEAADVAAFRQSLREHHARVALAAA<br>HSLSPHDTLSDPALDLLGLMRAGGLILRFEGRWQTLGEVPPAP<br>AVDALLAWLETQPGALVQTDALGQLWPAGADLAPSAAGLLAI<br>SVGEGWSECLVWLRPELRLEVAWGGATPDQAKDDLGPRHSFD<br>TYLEEKRGYAEPWHPGEIEEAQDLRDTLTGALGEAEAAAKEA<br>AAKEAAAKEAAAKANKCGQRSKFGINRPAVLAPEDGLAMSLH<br>FMTLGGSSLSPTEGKGSGLQGHIMENPQYFSDTCVHHIKRQDII<br>LKWELGEGAFGKVFLAECYNLLNDQDKMLVAVKALKETSEN<br>ARQDFHREAELLTMLQHQHIVRFFGVCTEGGPLLMVFEYMRH<br>GDLNRFLRSHGPDAKLLAGGEDVAPGPLGLGQLLAVASQVAA<br>GMVYLASLHFVHRDLATRNCLVGQGLVVKIGDFGMSRDIYST<br>DYYRVGGRTMLPIRWMPPESILYRKFSTESDVWSFGVVLWEIF<br>TYGKQPWYQLSNTEAIECITQGRELERPRACPPDVYAIMRGCW<br>QREPQQRLSMKDVHARLQALAQAPPSYLDVLG
cyto-Dr-TrkB	MSRDPLPFFPPLYLGGPEITTENCEREPIHIPGSIQPHGALLTADG<br>HSGEVLQMSLNAATFLGQEPTVLRGQTLAALLPEQWPALQAA<br>LPPGCPDALQYRATLDWPAAGHLSLTVHRVGELLILEFEPTEA<br>WDSTGPHALRNAMFALESAPNLRALAEVATQTVRELTGFDRV<br>MLYKFAPDATGEVIAEARREGLHAFLGHRFPASDIPAQARALY<br>TRHLLRLTADTRAAAVPLDPVLNPQTNAPTPLGGAVLRATSPM<br>HMQYLRNMGVGSSLSVSVVVGGQLWGLIACHHQTPYVLPPDL<br>RTTLEYLGRLLSLQVQVKEAADVAAFRQSLREHHARVALAAA<br>HSLSPHDTLSDPALDLLGLMRAGGLILRFEGRWQTLGEVPPAP<br>AVDALLAWLETQPGALVQTDALGQLWPAGADLAPSAAGLLAI<br>SVGEGWSECLVWLRPELRLEVAWGGATPDQAKDDLGPRHSFD<br>TYLEEKRGYAEPWHPGEIEEAQDLRDTLTGALGEAEAAAKEA<br>AAKEAAAKEAAAKAKLARHSKFGMKGPASVISNDDDSASPLH<br>HISNGSNTPSSSEGGPDAVIIGMTKIPVIENPQYFGITNSQLKPDT<br>FVQHIKRHNIVLKRELGEGAFGKVFLAECYNLCPEQDKILVAV<br>KTLKDASDNARKDFHREAELLTNLQHEHIVKFYGVCVEGDPLI<br>MVFEYMKHGDLNKFLRAHGPDAVLMAEGNPPTELTQSQMLHI<br>AQQIAAGMVYLASQHFVHRDLATRNCLVGENLLVKIGDFGMS<br>RDVYSTDYYRVGGHTMLPIRWMPPESIMYRKFTTESDVWSLG<br>VVLWEIFTYGKQPWYQLSNNEVIECITQGRVLQRPRTCPQEVY<br>ELMLGCWQREPHTRKNIKNIHTLLQNLAKASPVYLDILG
DrBphP-PCM-TrkB	MGCIKSKRKDALYKEFSAGSAGSAGMSRDPLPFFPPLYLGGPEI<br>TTENCEREPIHIPGSIQPHGALLTADGHSGEVLQMSLNAATFLG

	QEPTVLRGQTLAALLPEQWPALQAALPPGCPDALQYRATLDW<br>PAAGHLSLTVHRVGELLILEFEPTEAWDSTGPHALRNAMFALE<br>SAPNLRALAEVATQTVRELTGFDRVMLYKFAPDATGEVIAEAR<br>REGLHAFLGHRFPASDIPAQARALYTRHLLRLTADTRAAAVPL<br>DPVLNPQTNAPTPLGGAVLRATSPMHMQYLRNMGVGSSLSVS<br>VVVGGQLWGLIACHHQTPYVLPPDLRTTLEYLGRLLSLQVQVK<br>EAADVAAFRQSLREHHARVALAAAHSLSPHDTLSDPALDLLGL<br>MRAGGLILRFEGRWQTLGEVPPAPAVDALLAWLETQPGALVQ<br>TDALGQLWPAGADLAPSAAGLLAISVGEGWSECLVWLRPELR<br>LEVAWGGATPDQAKDDLGPRHSFDTYLEEKRGYAEPWHPGEI<br>EEAQDLRDTLTGALGEKLARHSKFGMKGPASVISNDDDSASPL<br>HHISNGSNTPSSSEGGPDAVIIGMTKIPVIENPQYFGITNSQLKPD<br>TFVQHIKRHNIVLKRELGEGAFGKVFLAECYNLCPEQDKILVAV<br>KTLKDASDNARKDFHREAELLTNLQHEHIVKFYGVCVEGDPLI<br>MVFEYMKHGDLNKFLRAHGPDAVLMAEGNPPTELTQSQMLHI<br>AQQIAAGMVYLASQHFVHRDLATRNCLVGENLLVKIGDFGMS<br>RDVYSTDYYRVGGHTMLPIRWMPPESIMYRKFTTESDVWSLG<br>VVLWEIFTYGKQPWYQLSNNEVIECITQGRVLQRPRTCPQEVY<br>ELMLGCWQREPHTRKNIKNIHTLLQNLAKASPVYLDILG
DrBphP-PCM-DHp-<br>TrkB	MGCIKSKRKDALYKEFSAGSAGSAGMSRDPLPFFPPLYLGGPEI<br>TTENCEREPIHIPGSIQPHGALLTADGHSGEVLQMSLNAATFLG<br>QEPTVLRGQTLAALLPEQWPALQAALPPGCPDALQYRATLDW<br>PAAGHLSLTVHRVGELLILEFEPTEAWDSTGPHALRNAMFALE<br>SAPNLRALAEVATQTVRELTGFDRVMLYKFAPDATGEVIAEAR<br>REGLHAFLGHRFPASDIPAQARALYTRHLLRLTADTRAAAVPL<br>DPVLNPQTNAPTPLGGAVLRATSPMHMQYLRNMGVGSSLSVS<br>VVVGGQLWGLIACHHQTPYVLPPDLRTTLEYLGRLLSLQVQVK<br>EAADVAAFRQSLREHHARVALAAAHSLSPHDTLSDPALDLLGL<br>MRAGGLILRFEGRWQTLGEVPPAPAVDALLAWLETQPGALVQ<br>TDALGQLWPAGADLAPSAAGLLAISVGEGWSECLVWLRPELR<br>LEVAWGGATPDQAKDDLGPRHSFDTYLEEKRGYAEPWHPGEI<br>EEAQDLRDTLTGALGERLSVIRDLNRALTQSNAEWRQYGFVIS<br>HHMQEPVRLISQFAELLTRQPRAQDGSPDSPQTERITGFLLRETS<br>RLRSLTQDLHTYTALLSAPPPKLARHSKFGMKGPASVISNDDDS<br>ASPLHHISNGSNTPSSSEGGPDAVIIGMTKIPVIENPQYFGITNSQ<br>LKPDTFVQHIKRHNIVLKRELGEGAFGKVFLAECYNLCPEQDKI<br>LVAVKTLKDASDNARKDFHREAELLTNLQHEHIVKFYGVCVE<br>GDPLIMVFEYMKHGDLNKFLRAHGPDAVLMAEGNPPTELTQS<br>QMLHIAQQIAAGMVYLASQHFVHRDLATRNCLVGENLLVKIG<br>DFGMSRDVYSTDYYRVGGHTMLPIRWMPPESIMYRKFTTESD<br>VWSLGVVLWEIFTYGKQPWYQLSNNEVIECITQGRVLQRPRTC<br>PQEVYELMLGCWQREPHTRKNIKNIHTLLQNLAKASPVYLDIL<br>G
PDZ-Dr-TrkA	MAKQEIRVRVEKDPELGFSISGGVGGRGNPFRPDDDGIFVTRVQ<br>PEGPASKLLQPGDKIIQANGYSFINIEHGQAVSLLKTFQNTVELII<br>VREVSAGGSAGGSAGGAKQEIRVRVEKDPELGFSISGGVGGRG

	NPFRPDDDGIFVTRVQPEGPASKLLQPGDKIIQANGYSFINIEHG<br>QAVSLLKTFQNTVELIIVREVSRGEEDNMAIIKEFMRFKVHMEG<br>SVNGHEFEIEGEGEGRPYEGTQTAKLKVTKGGPLPFAWDILSPQ<br>FMYGSKAYVKHPADIPDYLKLSFPEGFKWERVMNFEDGGVVT<br>VTQDSSLQDGEFIYKVKLRGTNFPSDGPVMQKKTMGWEASSER<br>MYPEDGALKGEIKQRLKLKDGGHYDAEVKTTYKAKKPVQLPG<br>AYNVNIKLDITSHNEDYTIVEQYERAEGRHSTGGMDELYKEFSA<br>GSAGMSRDPLPFFPPLYLGGPEITTENCEREPIHIPGSIQPHGALLT<br>ADGHSGEVLQMSLNAATFLGQEPTVLRGQTLAALLPEQWPALQ<br>AALPPGCPDALQYRATLDWPAAGHLSLTVHRVGELLILEFEPTE<br>AWDSTGPHALRNAMFALESAPNLRALAEVATQTVRELTGFDRV<br>MLYKFAPDATGEVIAEARREGLHAFLGHRFPASDIPAQARALYT<br>RHLLRLTADTRAAAVPLDPVLNPQTNAPTPLGGAVLRATSPMH<br>MQYLRNMGVGSSLSVSVVVGGQLWGLIACHHQTPYVLPPDLR<br>TTLEYLGRLLSLQVQVKEAADVAAFRQSLREHHARVALAAAHS<br>LSPHDTLSDPALDLLGLMRAGGLILRFEGRWQTLGEVPPAPAVD<br>ALLAWLETQPGALVQTDALGQLWPAGADLAPSAAGLLAISVGE<br>GWSECLVWLRPELRLEVAWGGATPDQAKDDLGPRHSFDTYLE<br>EKRGYAEPWHPGEIEEAQDLRDTLTGALGEAEAAAKEAAAKEA<br>AAKEAAAKANKCGQRSKFGINRPAVLAPEDGLAMSLHFMTLG<br>GSSLSPTEGKGSGLQGHIMENPQYFSDTCVHHIKRQDIILKWELG<br>EGAFGKVFLAECYNLLNDQDKMLVAVKALKETSENARQDFHR<br>EAELLTMLQHQHIVRFFGVCTEGGPLLMVFEYMRHGDLNRFLR<br>SHGPDAKLLAGGEDVAPGPLGLGQLLAVASQVAAGMVYLASL<br>HFVHRDLATRNCLVGQGLVVKIGDFGMSRDIYSTDYYRVGGRT<br>MLPIRWMPPESILYRKFSTESDVWSFGVVLWEIFTYGKQPWYQL<br>SNTEAIECITQGRELERPRACPPDVYAIMRGCWQREPQQRLSMK<br>DVHARLQALAQAPPSYLDVLG

Myristoylation signal is highlighted in blue. Inserted artificial α-helical linkers are highlighted in grey. SAG linker is highlighted in yellow. mCherry sequence is highlighted in red.

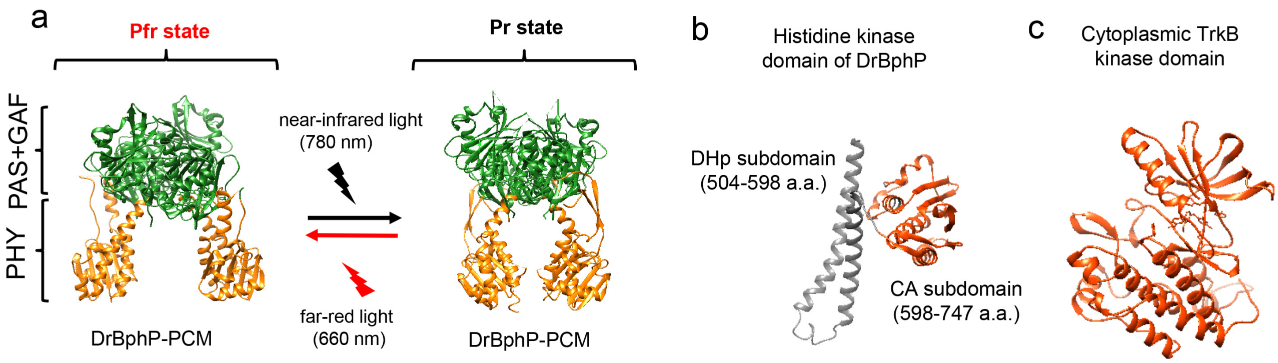

Supplementary Figure 1. Structural modules of DrBphP and Trk proteins used to design opto-kinases. (a) Light-induced changes in DrBphP-PCM protein upon FR (660 nm) and NIR (780 nm) light. Left is the Pfr state (PDB ID: 4O01), and right is the Pr state (PDB ID: 4O0P). (**b**) Structure of HK domain of DrBphP modelled on the basis of HK kinase of *Thermotoga maritima* (PDB ID:4JAV). **(c)** Structure of kinase domain of TrkB (PDB ID: 4ASZ).

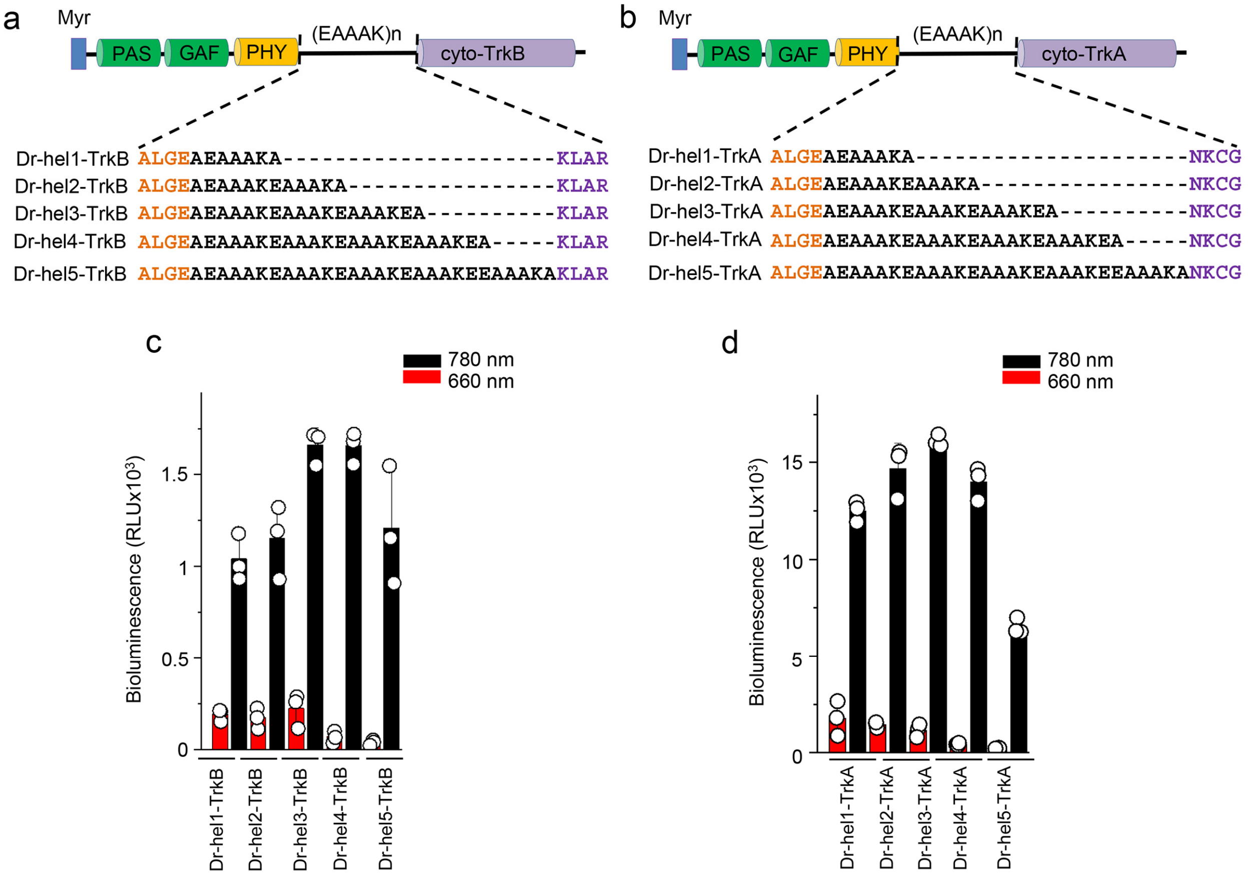

Supplementary Figure 2. Design and screening DrBphP-PCM-cyto-Trk fusions with rigid α-helical linkers of different length. (**a**) Schematically depicted structures of DrBphP-PCM-cyto-TrkB constructs containing a linker with different number of α-helical (hel) repeats -EAAAK-. (**b**) The same as in (a) but for DrBphP-PCM-cyto-TrkA constructs. (**c**) Luciferase assay for Elk-1-dependent transcription in PC6-3 cells expressing DrBphP-PCM-cyto-TrkB constructs shown in (a). PC6-3 cells were co-transfected with pCMVd2-DrBphP-PCM-cyto-TrkB-variant, pFR-Luc and pFA-Elk-1 plasmid mixture (mass ratio 1:100:5) for 6 h, after that medium was replaced with that without serum. Cells were grown for additional 30 h under 780 nm or 660 nm light (both 0.5 Mw cm^{-2}), lysed and analyzed for luciferase activity. (**d**) The same as in (c) but for DrBphP-PCM-cyto-TrkA-variants. Error bars represent s.d., n=3 experiments.

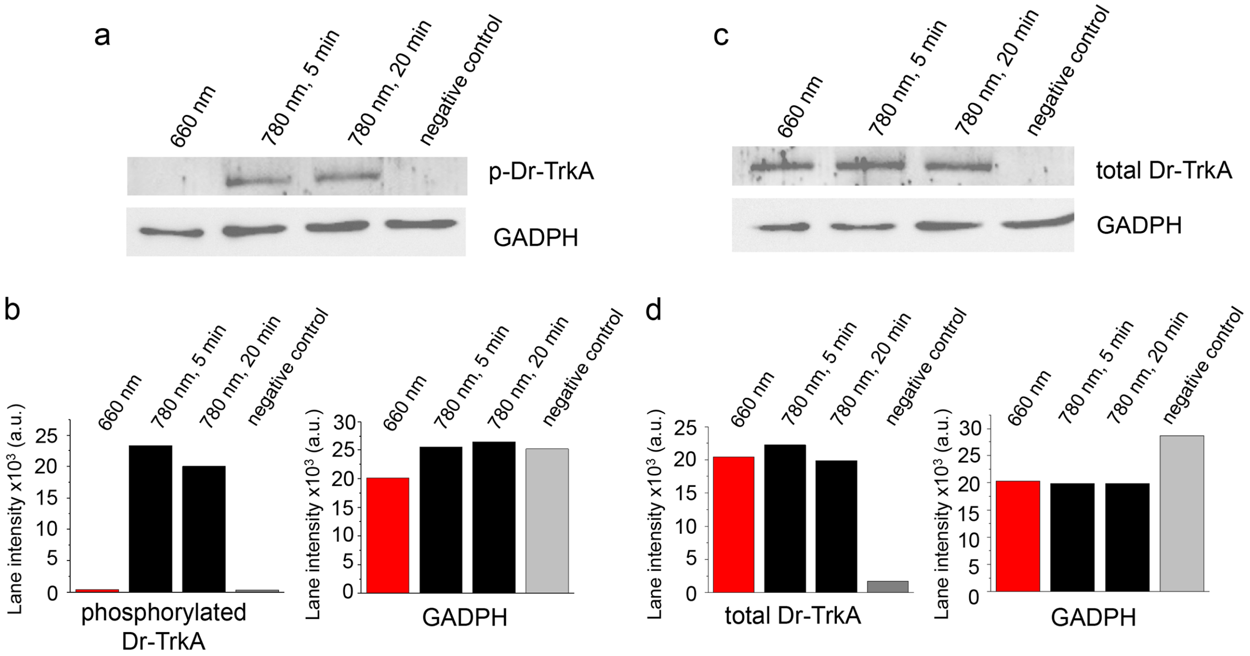

Supplementary Figure 3. Induction of Dr-TrkA phosphorylation with near-infrared light. (a) Western blot of phosphorylated Dr-TrkA and GADPH in HeLa cells expressing Dr-TrkA. HeLa cells were transfected with Dr-TrkA, then 6 h later were illuminated with 660 nm light for 18 h before induction with 780 nm light for 5 min and 20 min, respectively. Cells were lysed, proteins were separated by SDS-PAGE, transferred to nitrocellulose membrane, and probed by the respective antibodies (**b**) Quantification of the lane intensities in panel (a). Negative control corresponds to mock-transfected cells. (**c**) Western blot of total Dr-TrkA and GADPH in the same samples as in panel (a). (**d**) Quantification of the lane intensities in panel (c). Negative control corresponds to mock-transfected cells.

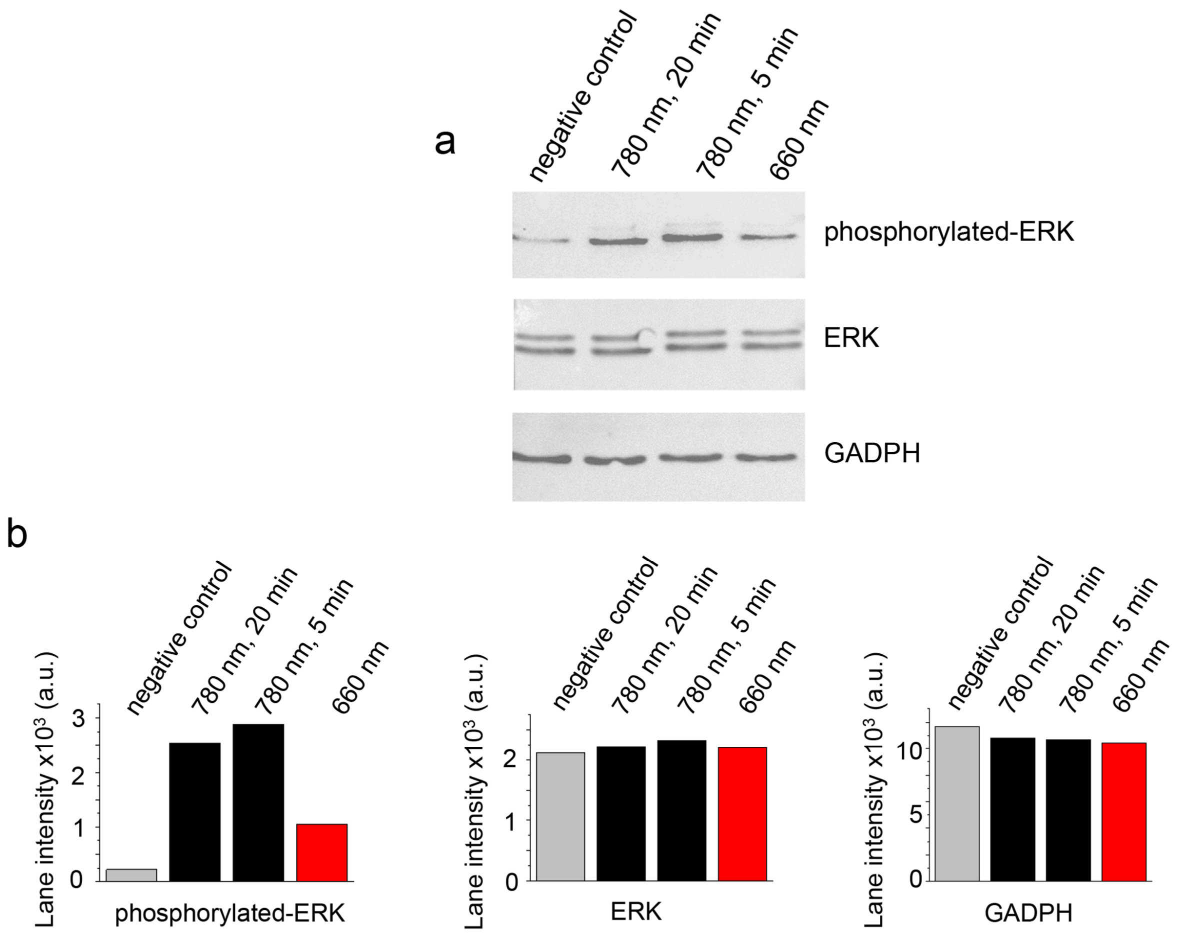

Supplementary Figure 4. Induction of ERK phosphorylation with near-infrared light. (a) Western blot of phosphorylated ERK, total ERK and GADPH in HeLa cells expressing Dr-TrkA. HeLa cells were transfected with Dr-TrkA, then 6 h later were illuminated with 660 nm light for 18 h before induction with 780 nm light for 5 min and 20 min, respectively. Cells were lysed, proteins were separated by SDS-PAGE, transferred to nitrocellulose membrane, and probed by anti-p-ERK rabbit antibodies (top panel). After p-ERK detection, the same membrane was stripped and re-probed with anti-ERK rabbit antibodies. After that, the same membrane was stripped and re-probed with mouse anti-GADPH antibodies. From left to right, mock-transfected cells, Dr-TrkA-expressing cells induced with 780 nm light for 20 min or 5 min, and Dr-TrkA-expressing cells kept under 660 nm light. **(b)** Quantification of the lane intensities in panel (a).

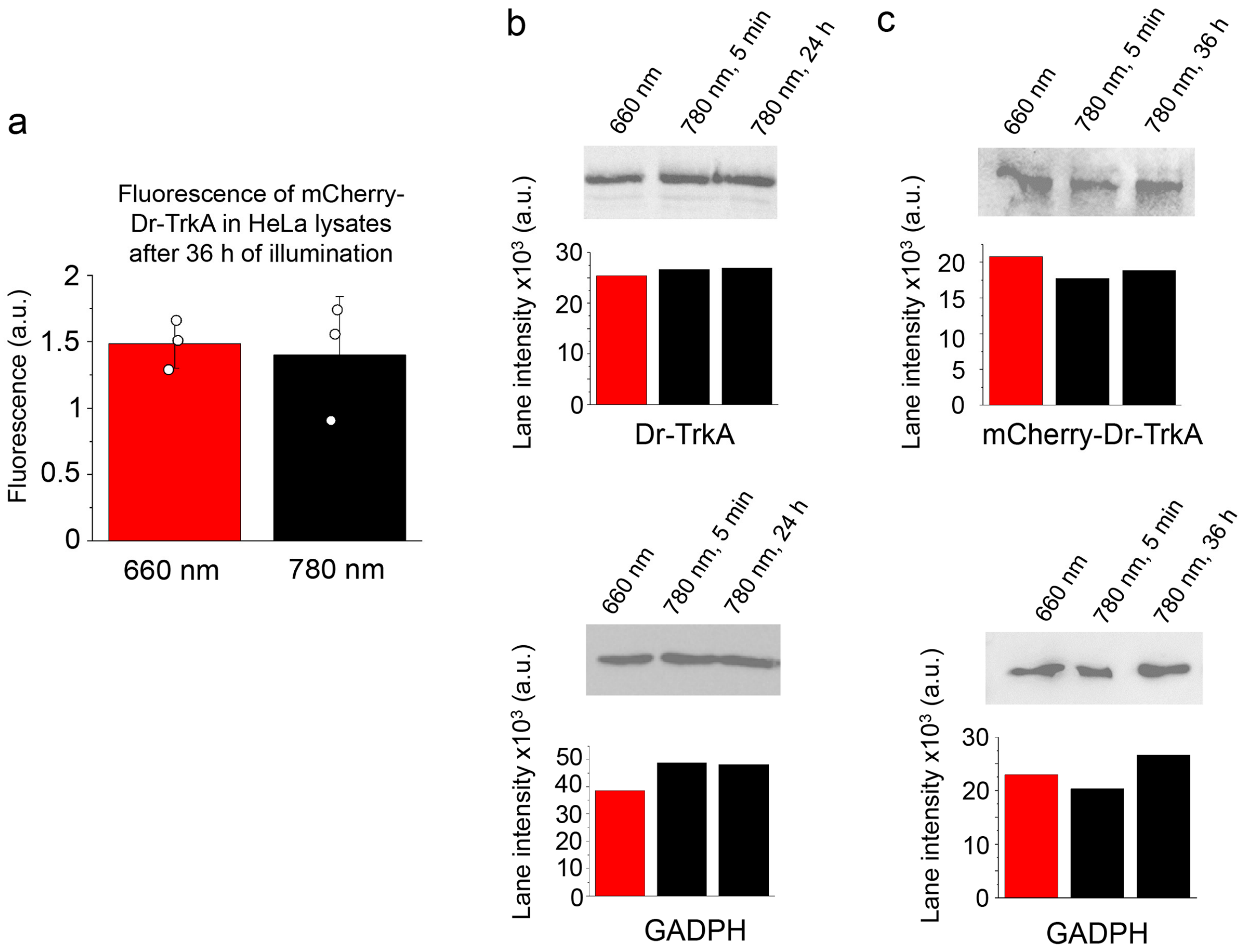

Supplementary Figure 5. Effect of far-red and near-infrared light on stability of Dr-TrkA. **(a)** HeLa cells were transfected with pcDNA-mCherry-Dr-TrkA and kept under either 660 nm or 780 nm light for 36 h. Then the cells were lysed, and mCherry fluorescence intensity in lysates was quantified with a Victor X5 plate reader using ex. 560 nm and em. 590 nm wavelengths. Error bars represent s.d., n=3 experiments. **(b)** Top: Western blot of total Dr-TrkA in HeLa cells, transfected with pcDNA-Dr-TrkA plasmid, kept for 24 h under either 660 nm or 780 nm light. Left to right: 660 nm illuminated cells, induced for 5 min with 780 nm light cells, and 780 nm illuminated cells, respectively. Quantification of the lane intensities is presented below. Bottom: Western blot of the same membrane stripped and re-probed with anti-GADPH antibodies. Quantification of the lane intensities is presented below. **(c)** Top: Western blot of total Dr-TrkA in HeLa cells transfected with pcDNA-mCherry-Dr-TrkA plasmid, kept for 36 h under either 660 nm or 780 nm light. Left to right: 660 nm illuminated cells, induced for 5 min with 780 nm light cells, and 780 nm illuminated cells, respectively. Quantification of the lane intensities is presented below. Bottom: Western blot of the same membrane stripped and re-probed with anti-GADPH antibodies. Quantification of the lane intensities is presented below. Light-dependent proteolysis was not observed for either Dr-TrkA or mCherry-Dr-TrkA proteins.

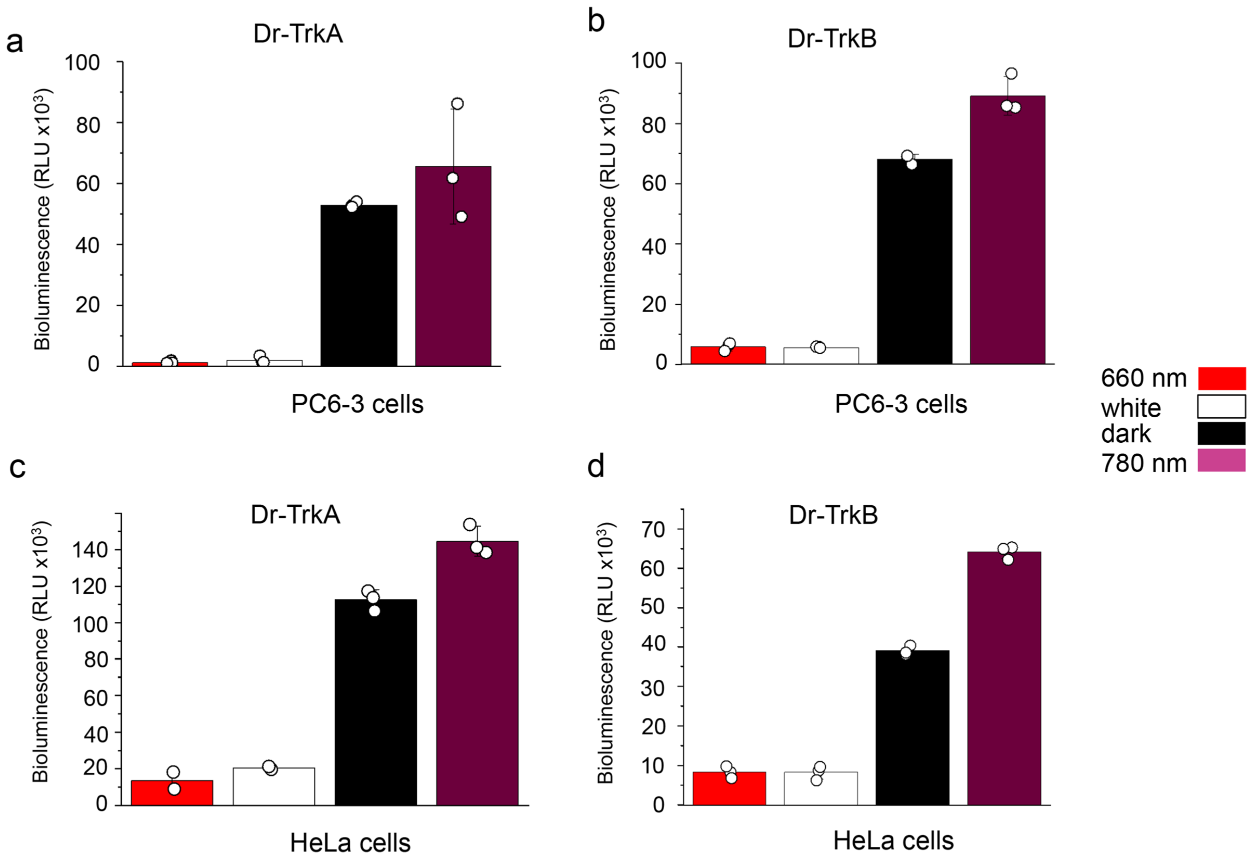

Supplementary Figure 6. **Effect of white, far-red and near-infrared light or darkness on Dr-Trk activation. (a,b)** PC6-3 and **(c,d)** HeLa cells were transfected with either Dr-TrkA (a,c) or Dr-TrkB (b,d) constructs, grown for 36 h under the respective illumination or in darkness, lysed and analyzed for luciferase expression from the Elk-1-dependent promoter (see Fig. 1c). Error bars represent s.d., n=3 experiments.

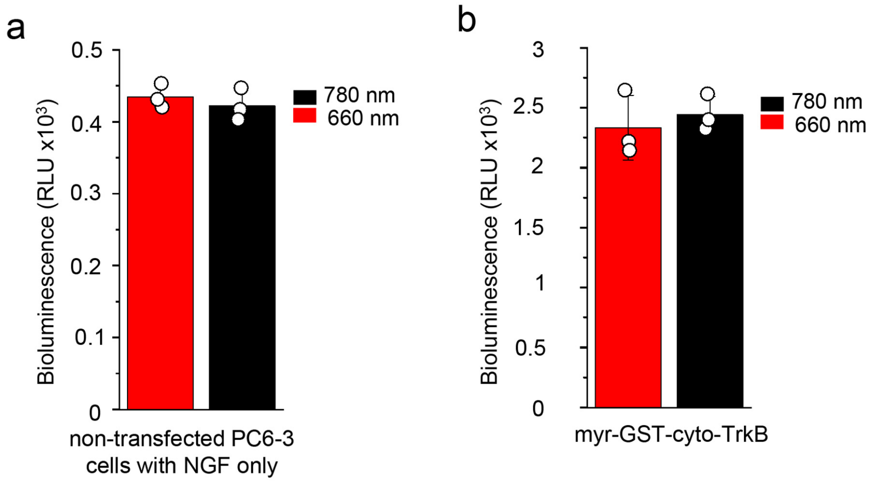

Supplementary Figure 7. Light-independent regulation of MAPK/ERK signaling in PC6-3 cells. (a) Luciferase assay of Elk-1-driven transcription in non-transfected PC6-3 cells grown for 30 h in presence of 100 ng/ml NGF under 660 nm or 780 nm light. (**b)** Luciferase assay of Elk-1-driven transcription in PC6-3 cells expressing myr-GST-TrkB, grown under 660 nm or 780 nm light, and analysed for luciferase activity. Error bars represent s.d., n=3 experiments.

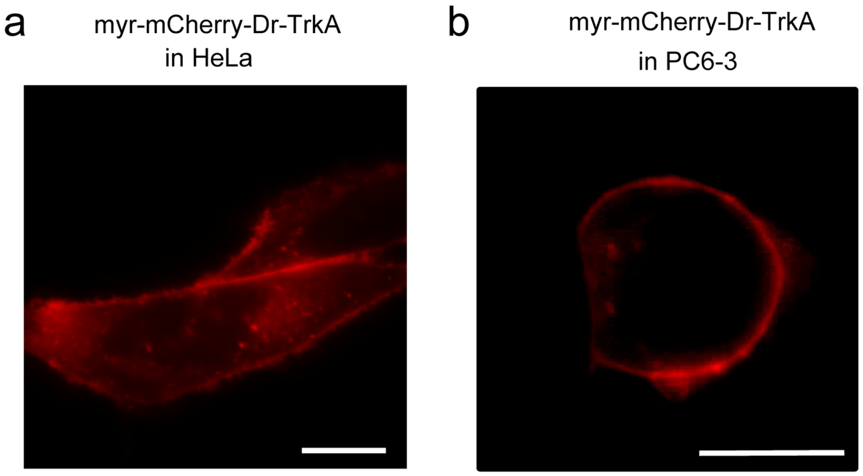

Supplementary Figure 8. Membrane localization of myristoylated Dr-TrkA. (a) Epifluorescence image of HeLa cells transfected with pMyr-mCherry-Dr-TrkA plasmid. Scale bar, 10 µm. (**b**) Epifluorescence image of PC6-3 cells transfected by pMyr-mCherry-Dr-TrkA plasmid. Scale bar, 10 µm.

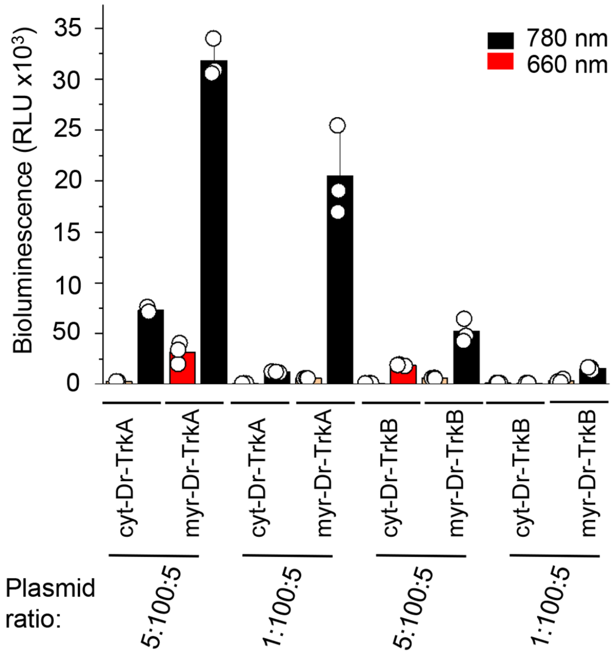

Supplementary Figure 9. Activity of cytoplasmic cyt-Dr-Trks and membrane-bound myr-Dr-Trks. Two Dr-Trk, pFr-Luc and pFA-Elk-1 plasmid ratios (5 ng Dr-Trk : 100 ng pFR-Luc : 5 ng pFA-Elk-1, and 1 ng Dr-Trk : 100 ng pFR-Luc : 5 ng pFA-Elk-1) were tested for cytoplasmic and membrane-bound constructs of both Dr-TrkA and Dr-TrkB. The co-transfected PC6-3 were grown under 660 nm or 780 nm light and analysed for luciferase activity. Error bars represent s.d., n=3 experiments.

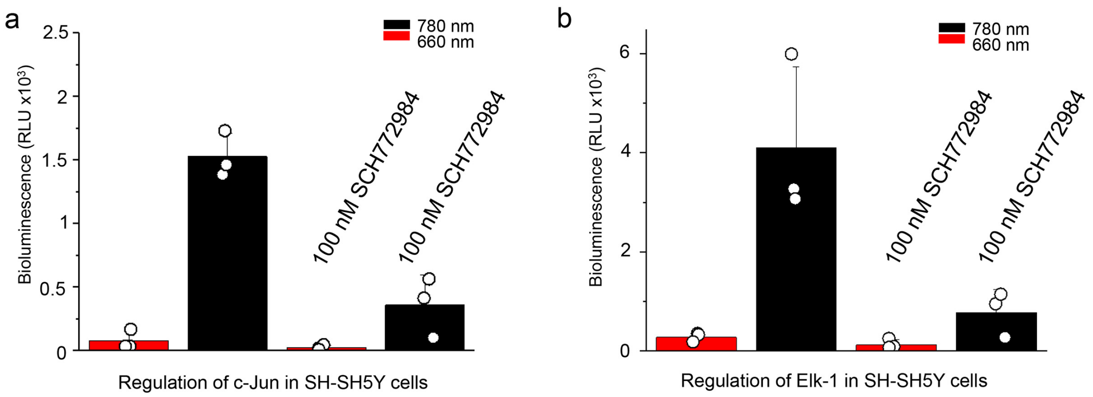

Supplementary Figure 10. Effect of ERK inhibitor SCH772984 on c-Jun and Elk-1 activation. SH-SH5Y cells were transfected with Dr-TrkA construct for 36 h, lysed and analyzed by luciferase assay. **(a)** c-Jun –dependent luciferase activity in the cells transfected by Dr-TrkA. The cells were kept under 660 nm and 780 nm light ether without or with SCH772984 inhibitor. **(b)** Elk-1 –dependent luciferase activity in the cells transfected by Dr-TrkA. The cells were kept under 660 nm and 780 nm light without or with SCH772984 inhibitor. Error bars represent s.d., n=3 experiments.

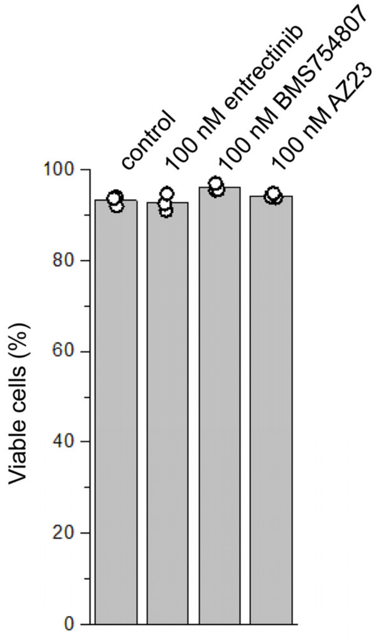

Supplementary Figure 11. Effect of inhibitors on viability of PC6-3 cells. Cells were grown for 30 h in the presence of 100 nM of either entrectinib, BMS754807 or AZ23, detached, stained with propidium iodide, and analyzed using flow cytometry. The viable cells for each condition represent a fraction of the propidium iodide-negative cells in the population. Error bars represent s.d., n=3 experiments.

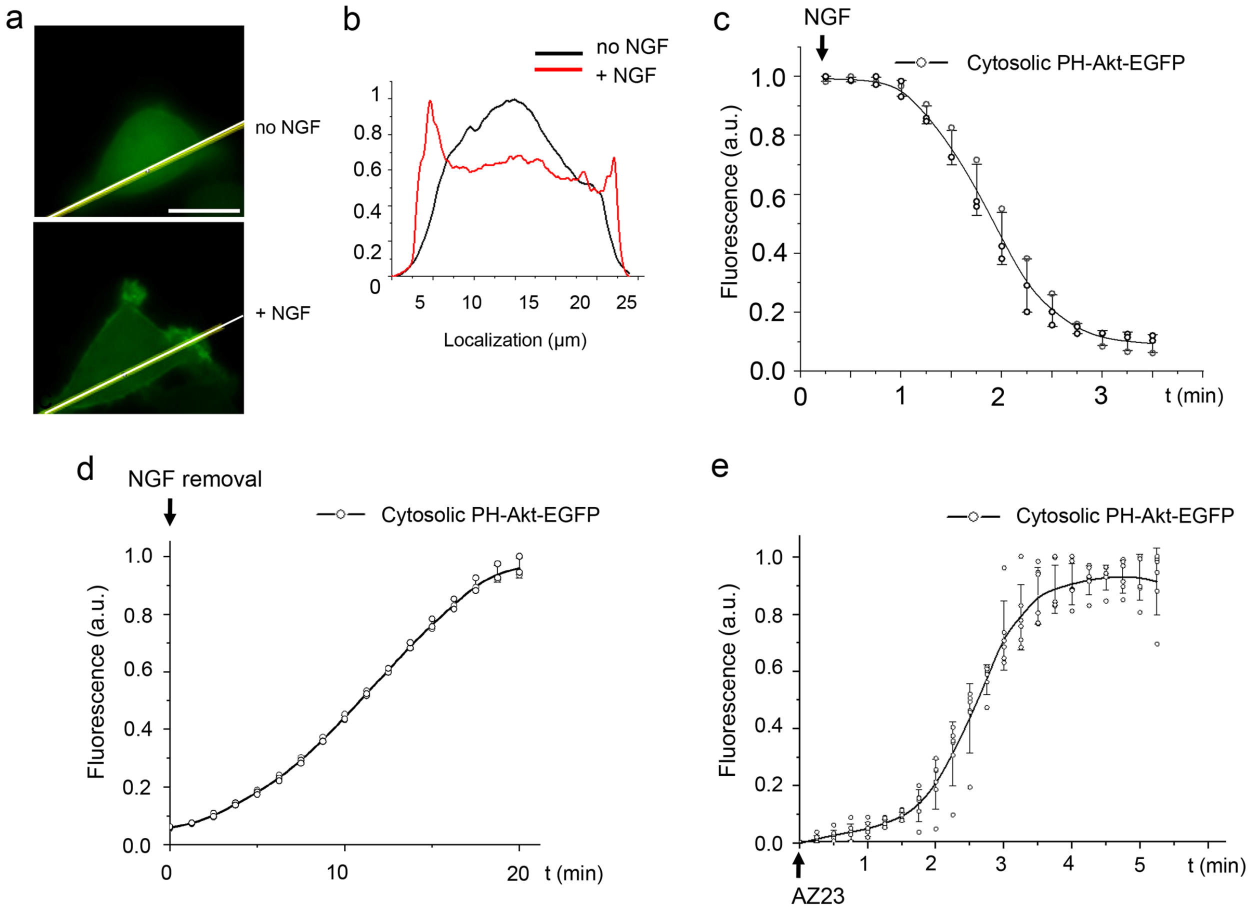

Supplementary Figure 12. Regulation of PI3K activity by NGF detected by PH-Akt-EGFP relocalization from cytosol to plasma membrane. (**a**) Epifluorescence images of PC6-3 cells expressing PH-Akt-EGFP reporter before (top) and 10 min after addition of 50 ng/ml NGF (bottom). Scale bar, 10 µm. **(b)** Epifluorescence intensity profile of PC6-3 cells expressing PH-Akt-EGFP fluorescence before (black line) and after (red line) addition of 50 ng/ml NGF. (**c**) Relative decrease of cytosolic PH-Akt-EGFP fluorescence of PC6-3 cells after addition of 50 ng/ml NGF. (**d**) Relative increase of cytosolic PH-Akt-GFP fluorescence in PC6-3 after NGF removal. (**e**) Relative increase of cytosolic PH-Akt-EGFP fluorescence of PC6-3 cells after addition of the Trk's specific inhibitor AZ23 (100 nM). Error bars represent s.d., n=3 experiments.

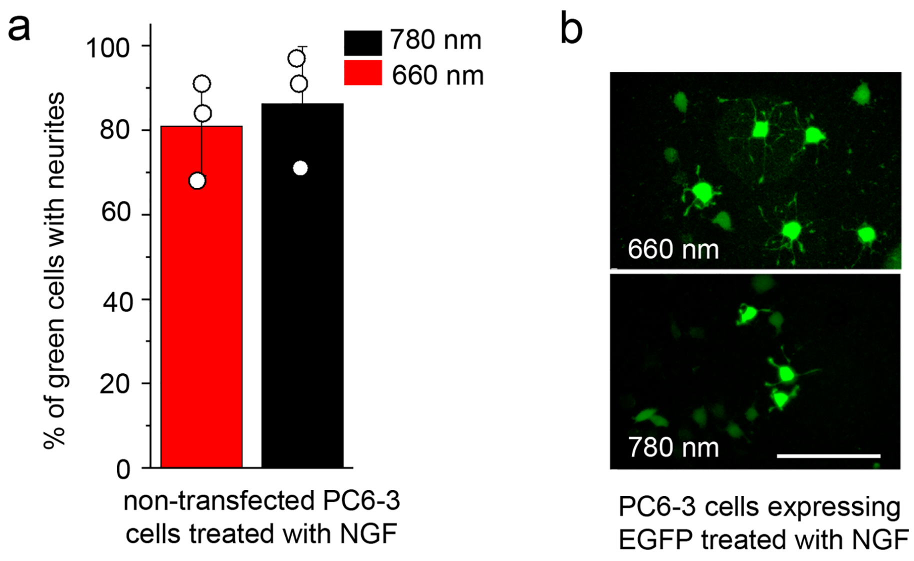

Supplementary Figure 13. Neurite growth in NGF treated PC6-3 cells. (**a**) Quantification of PC6-3 cells bearing neurites grown for 24 h in presence of 50 ng/ml NGF under 660 nm or 780 nm light. (**b**) Epifluorescence images of PC6-3 cells transfected with pEGFP-C1 and grown for 24 h in presence of 50 ng/ml NGF under 660 nm or 780 nm light. Scale bar, 100 µm. Error bars represent s.d., n=3 experiments.

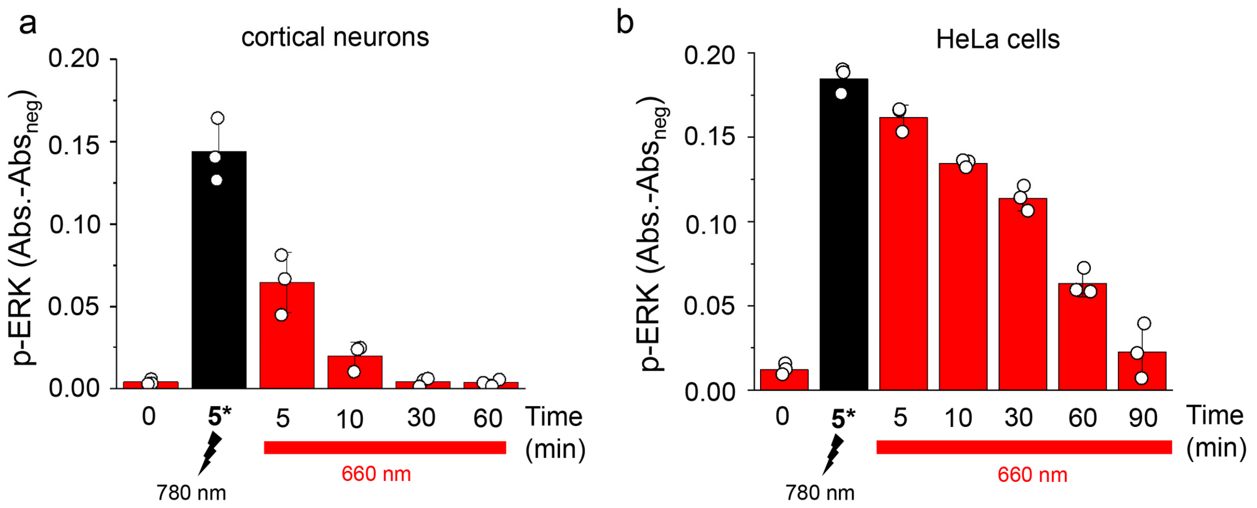

Supplementary Figure 14. Reversible activation of ERK pathway by Dr-TrkA in cortical neurons and HeLa cells. (a) Reversible accumulation of phospho-ERK in cultured neurons expressing Dr-TrkA. Neurons were transduced with AAV9 encoding Dr-TrkA construct and kept under 660 nm light. After that, cells were activated by 5 min pulse of 780 nm light and again kept under 660 nm light. At each time point, cells were fixed, permeabilized, probed with anti-phospho-ERK rabbit antibodies and goat anti-rabbit HRP conjugate, and incubated with TMB-ELISA substrate. From left to right: cells kept under 660 nm light, cells illuminated for 5 min with 780 nm light, cells gradually inactivated under 660 nm light for 5, 10, 30 and 60 min. **(b)** Reversible accumulation of phospho-ERK in HeLa cells expressing Dr-TrkA. Cells were transfected with Dr-TrkA construct and kept under 660 nm light. The other procedures were similar to (a). Error bars represent s.d., n=3 experiments.

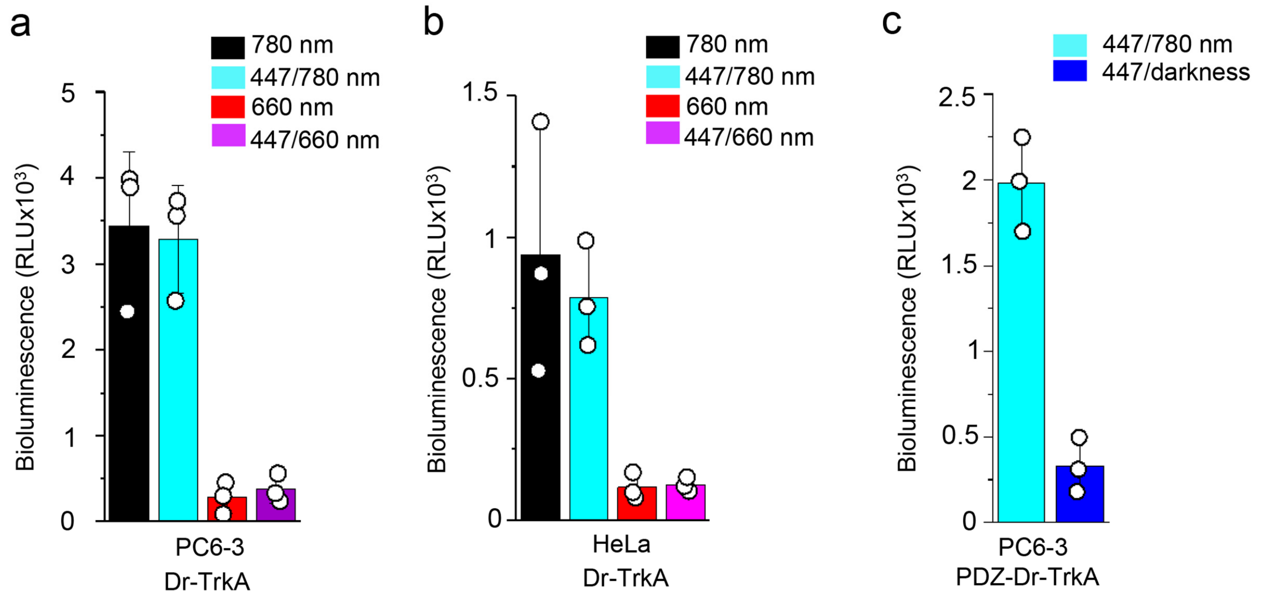

Supplementary Figure 15. Action of combination of 780 nm with 470 nm light on Dr-TrkA activity. Luciferase assay of the Elk-1-driven transcription was used (see **Fig. 1c**). (**a**) PC6-3 and (**b**) HeLa cells co-transfected with pDr-TrkA, pFR-Luc, and pFA-Elk-1 plasmids. From left to right: cells were grown under 660 nm light, 780 nm light, 30 s pulse of 447 nm light followed by 5 s pulse of 780 nm light, or 30 s pulse of 447 nm light followed by 5 s pulse of 660 nm light. All light intensities were 0.3 mW cm^{-2}. (**c**) Stimulation of PC6-3 cells co-transfected with pPDZ-Dr-TrkA, pLOVpep-stargazin, pFR-Luc, and pFA-Elk-1 plasmids illuminated with constant 447 nm and constant 780 nm light together or with constant 447 nm light only. Likely, 447 nm light alone photoconverts Dr-TrkA from Pr to Pfr state via a minor absorption band at ~400 nm common for bacterial phytochromes, called Soret band, which corresponds to the absorption of individual pyrrole rings of BV. Error bars represent s.d., n=3 experiments.
